# Antigen mobility regulates the dynamics and precision of antigen capture in the B cell immune synapse

**DOI:** 10.1073/pnas.2422528122

**Published:** 2025-05-12

**Authors:** Hannah C. W. McArthur, Anna T. Bajur, Maro Iliopoulou, Katelyn M. Spillane

**Affiliations:** ^a^Department of Physics, King’s College London, London WC2R 2LS, United Kingdom; ^b^Randall Centre for Cell and Molecular Biophysics, King’s College London, London SE1 1UL, United Kingdom; ^c^Department of Life Sciences, Imperial College London, London SW7 2AZ, United Kingdom

**Keywords:** B cell receptor, immune synapse, endocytosis, mechanobiology

## Abstract

B cells discriminate antigens in immune synapses by capturing them from antigen-presenting cells (APCs). This discrimination relies on the application of mechanical force to B cell receptor (BCR)-antigen bonds, allowing B cells to selectively disrupt low-affinity interactions while internalizing high-affinity antigens. Using DNA-based tension sensors combined with high-resolution imaging, we demonstrate that the magnitude, location, and timing of forces within the immune synapse are influenced by the fluidity of the antigen-presenting membrane. Transitioning antigens from a high-mobility to a low-mobility substrate significantly increases the probability and speed of antigen extraction while also improving affinity discrimination. This shift in antigen mobility also reshapes the synapse architecture, altering spatial patterns of antigen uptake. Despite these adaptations, B cells maintain consistent levels of proximal and downstream signaling pathway activation regardless of antigen mobility. They also efficiently transport internalized antigens to major histocompatibility complex class II (MHCII)-positive compartments for processing. These results demonstrate that B cells mount effective responses to antigens across diverse physical environments, though the characteristics of that environment may influence the speed and accuracy of B cell adaptation during an immune response.

## Significance

To generate protective antibodies, B cells must capture and discriminate antigens displayed on antigen-presenting cell (APC) membranes. While B cell receptor (BCR)-antigen binding affinity has long been considered the primary driver of this process, we show that a B cell–extrinsic factor—antigen mobility within the presenting membrane—plays a decisive role. Using DNA nanostructures on model membranes to precisely control antigen valency and mobility, we demonstrate that reduced antigen mobility enhances both the speed and selectivity of antigen extraction in the immune synapse. This finding reveals how APCs, by controlling the physical state of presented antigens, may tune the fidelity of B cell activation and shape the antibody repertoire.

Protective antibody responses begin when B cells capture antigens from antigen-presenting cells (APCs). Upon binding antigens through their B cell receptors (BCRs), B cells internalize and process these antigens, ultimately displaying antigenic peptides on major histocompatibility complex class II (MHCII) molecules (1, 2). This presentation elicits T cell help, which—combined with BCR-induced metabolic and transcriptional activation—drives B cell expansion and differentiation into plasma, memory, or germinal center cells (3–9). Through class switch recombination and somatic mutation, activated B cells generate diverse BCRs, establishing a pool of cells that produces broad and potent antibodies and maintains immunological memory (10). Without T cell help, however, antigen-stimulated B cells undergo apoptosis (11). Thus, a B cell’s fate hinges on its ability to internalize and present antigen.

Within secondary lymphoid organs, APCs display intact, multivalent antigens on their surfaces for B cells to surveil (12–16). Using actin-based membrane protrusions, B cells rapidly probe APC surfaces with their BCRs (17). This process typically lasts just five minutes when cognate antigen is absent (12, 15). Upon encountering cognate antigen, a B cell establishes a longer-lived immune synapse, characterized first by actin-driven spreading across the APC surface to maximize antigen binding, followed by coordinated actions of the actin and microtubule networks to sweep antigens from the synapse periphery to its center (18–20). During this centripetal movement, actomyosin structures generate pulling forces on BCR–antigen bonds, rupturing low-affinity interactions while extracting high-affinity antigens for internalization (21). This process selectively promotes the survival of high-affinity B cells (22), ultimately driving the production of high-affinity antibodies (23).

B cells use mechanical forces not only to extract membrane-presented antigens but also to sense physical properties of APCs. B cells engage APCs through a chain of noncovalently linked proteins including the BCR, antigen, tethering molecules (e.g., antibodies and complement), and APC receptors (e.g., Fc and complement receptors) (24, 25). Forces propagated from BCRs through these molecular connections create a tug-of-war that makes antigen acquisition sensitive to the relative strength of each binding interface (26). For example, when B cells pull on antigens firmly anchored to stiff APC membranes, they achieve precise affinity discrimination but limited antigen extraction (27). Conversely, weakly tethered antigens or soft APC membranes enhance extraction efficiency at the expense of affinity discrimination (27). Thus, the precision of B cell antigen capture depends not only on BCR–antigen bond affinity and mechanical resistance, but also on the physical properties of APCs and their antigen-anchoring molecules.

APC membranes vary not only in stiffness but also fluidity, which is governed by membrane viscosity and interactions between membrane molecules and the cortical cytoskeleton (28). While B cell activation studies often employ fluid planar lipid bilayers (PLBs) that permit free diffusion of tethered antigens (18, 29, 30), the receptors presenting antigens on actual APCs have significantly restricted mobility within the plasma membrane (31, 32). This difference in antigen mobility profoundly influences early events in B cell activation. High antigen mobility enhances BCR microcluster growth, signaling, and transport (30, 33), whereas low antigen mobility promotes cell spreading (33) and traction force generation (34, 35). Yet, the impact of antigen mobility on B cell antigen internalization remains unexplored. It is plausible that highly mobile antigens are preferentially internalized through enhanced activation of signaling pathways that assemble force-generating actomyosin structures (36). Alternatively, frictional coupling of low-mobility antigens to centripetal actin flow via the BCR may load higher forces that are more capable of wrenching antigens from their tethers (37, 38). Moreover, mobility-dependent force variations in the synapse could influence both the spatiotemporal dynamics of antigen extraction and the subsequent trafficking of internalized antigens to processing compartments.

To explore these possibilities, we used DNA nanostructures to create multivalent antigens and quantify their force-mediated extraction from model membranes of different viscosity. We hypothesized that increasing membrane viscosity, thereby reducing antigen mobility, would amplify forces on BCR–antigen bonds and enhance the efficiency and precision of antigen uptake. Our results strongly support this model and reveal an unexpected temporal advantage: Reduced antigen mobility accelerates these processes, enabling B cells to make activation decisions within physiologically relevant timescales. These findings suggest that APCs, by presenting immobilized, multivalent antigens, may actively shape antibody responses by promoting the rapid and selective activation of high-affinity B cells.

## Results

### Controlling Antigen Valency and Mobility with DNA Nanostructures and PLBs.

B cell activation outcomes are influenced by both the valency and mobility of antigens (33, 39, 40). To independently control these parameters, we combined antigen-coupled DNA nanostructures with glass-supported PLBs. DNA nanostructures provide precise control over the number of linked antigens, unlike protein scaffolds that offer only statistical control over antigen multimerization (41). Additionally, a defined number of fluorophores can be added to each DNA structure for quantitative analysis of antigen densities (42).

**Fig. 1.**
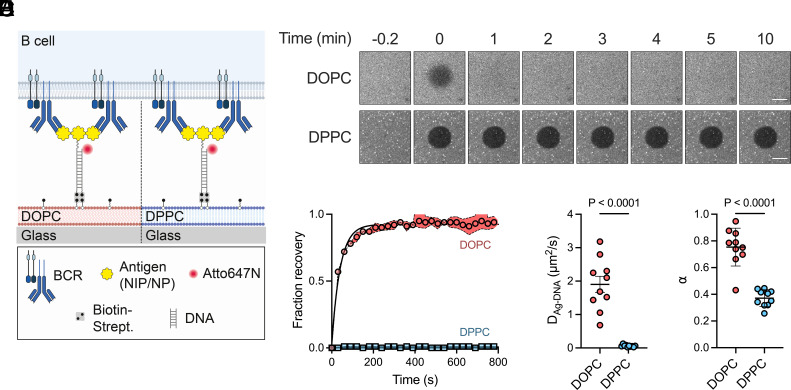
Changing antigen mobility by changing substrate viscosity. (*A*) Schematic showing BCR crosslinking by DNA-conjugated antigens tethered to glass-supported lipid bilayers. (*B*–*D*) Membrane fluidity characterization. (*B*) Time-lapse fluorescence images showing FRAP of labeled lipids. (Scale bars, 20 µm.) (*C*) FRAP recovery curves (mean ± SEM) from DOPC and DPPC bilayers, with a minimum of three regions analyzed per bilayer. (*D*) Diffusion coefficients and (*E*) anomalous diffusion exponents of antigen-DNA sensors on each bilayer type, determined by single-particle tracking. Each data point represents the mean value per bilayer (n = 10 bilayers per condition), calculated from 673 (DOPC) and 1,857 (DPPC) total trajectories. Bars represent mean ± SEM. *P* values were determined by two-tailed unpaired *t* test.

Each DNA structure was covalently bound to three copies of NIP (4-hydroxy-3-iodo-5-nitrophenyl), a hapten antigen that binds the BCR of primary naïve B1-8 B cells with a 3D K_d_ value of 0.33 µM (21) (Fig. 1*A*). The N-hydroxysuccinimide-functionalized haptens were attached to the DNA via Uni-Link modifications, featuring amino groups separated from the phosphodiester backbone by a four-carbon chain (*SI Appendix,* Fig. S1*A*). Due to the high conformational flexibility of these moieties, we computationally analyzed the structure to determine the mean interhapten distance, which ranged from <1 nm at equilibrium (*SI Appendix,* Fig. S1*B*) to 2.9 nm when fully extended (*SI Appendix,* Fig. S1*C*). Given that the IgM-class BCR cannot bind bivalently to antigens spaced closer than 3.6 nm (43), each trivalent antigen-DNA construct likely induces crosslinking of 2 to 3 BCRs.

To manipulate antigen mobility, we constructed PLBs using either DOPC or DPPC lipids. These lipids have identical head groups, maintaining a constant chemical composition for interfacing with cells, but different tail groups that alter lipid packing, resulting in surface viscosities of 8.4 × 10^–11^ Pa·s·m for DOPC and 3.0 × 10^–9^ Pa·s·m for DPPC (44). Fluorescence recovery after photobleaching (FRAP) measurements of PLBs doped with fluorescent lipids confirmed the contrasting viscosities of DOPC and DPPC bilayers (Fig. 1*B*). DOPC bilayers recovered to 95% with a half-time of 28 s, as expected for highly mobile lipids in a low-viscosity membrane, while DPPC bilayers recovered by just ~2%, indicative of a high-viscosity membrane that limits lipid movement (Fig. 1*C*).

To quantify the impact of bilayer viscosity on antigen mobility, we attached antigen-DNA structures to the PLBs using biotin–streptavidin interactions and measured their diffusion by single-particle tracking (*SI Appendix,* Fig. S2 *A* and *B* and Movies S1 and S2). Mean-squared displacement (MSD) analysis revealed that the diffusion constants were 1.9 ± 0.77 µm^2^/s on DOPC and 0.057 ± 0.025 µm^2^/s on DPPC (mean ± SD; Fig. 1*D* and *SI Appendix*, Fig. S2*C*), indicating that a 35-fold increase in PLB viscosity resulted in a 33-fold decrease in antigen-DNA mobility. This change agrees with a Stokes–Einstein-like expression (45) relating the diffusion constant, *D,* to the bilayer surface viscosity, $\eta_{m}$, as

$$D=\frac{k_{B}T\lambda }{4\pi \eta_{m}R},$$

where $k_{B}$ is the Boltzmann constant, T is the absolute temperature, λ is the characteristic length indicative of membrane perturbation (on the order of a single lipid, 0.5 nm) (44), R is the radius of the diffusing particle (46), and $\eta_{m}=\eta_{m}^{b}h$, where $\eta_{m}^{b}$ is the bulk viscosity of the bilayer and h the bilayer thickness (5.9 nm for DOPC and 6.3 nm for DPPC) (44, 47). For our experiments, we assume R to be the radius of two lipids (1 nm), as each antigen-DNA construct is doubly biotinylated and tethered to biotinylated lipids in the bilayer through a tetravalent streptavidin molecule. The anomalous diffusion exponents determined from a log–log plot of MSD versus time also revealed stronger confinement of DNA sensors on DPPC bilayers compared to DOPC (α = 0.75 ± 0.14 and 0.37 ± 0.065 for DOPC and DPPC, respectively; mean ± SD) (Fig. 1*E* and *SI Appendix*, Fig. S2D). Of note, the diffusion constant of antigen-DNA on the DPPC bilayer closely matches the mean diffusion constant of immune complexes on subcapsular sinus macrophage surfaces (0.011 µm^2^/s) (48). Thus, by altering bilayer viscosity, we can reliably influence antigen diffusion to mimic mobility characteristics that B cells encounter in vivo.

### High Substrate Viscosity Limits Antigen Binding but Enhances Cell Spreading and Signaling.

To investigate how bilayer viscosity—and consequently, antigen mobility—affects early B cell activation events, we stimulated B1-8 B cells using NIP_3_-DNA-coated PLBs. The NIP_3_-DNA density was adjusted to ~3,000 per µm^2^ on average (*SI Appendix,* Fig. S3 and Table S1), corresponding to ~9,000 NIP per µm^2^. This density likely approaches the upper limit experienced by B cells in vivo. For instance, follicular dendritic cells express ~300 complement receptor 2 molecules per µm^2^ that present multivalent antigens such as HIV virions (each expressing ~10 trimeric envelope proteins) which, if saturated, would present ~9,000 epitopes/µm^2^ (49, 50). For these experiments, we used a DNA duplex that cannot be ruptured by the forces B cells exert on BCR–antigen bonds. This resistance is achieved by anchoring both strands of the duplex to the bilayer via biotin–streptavidin interactions (Fig. 1*A* and *SI Appendix,* Table S2) (22, 51).

**Fig. 2.**
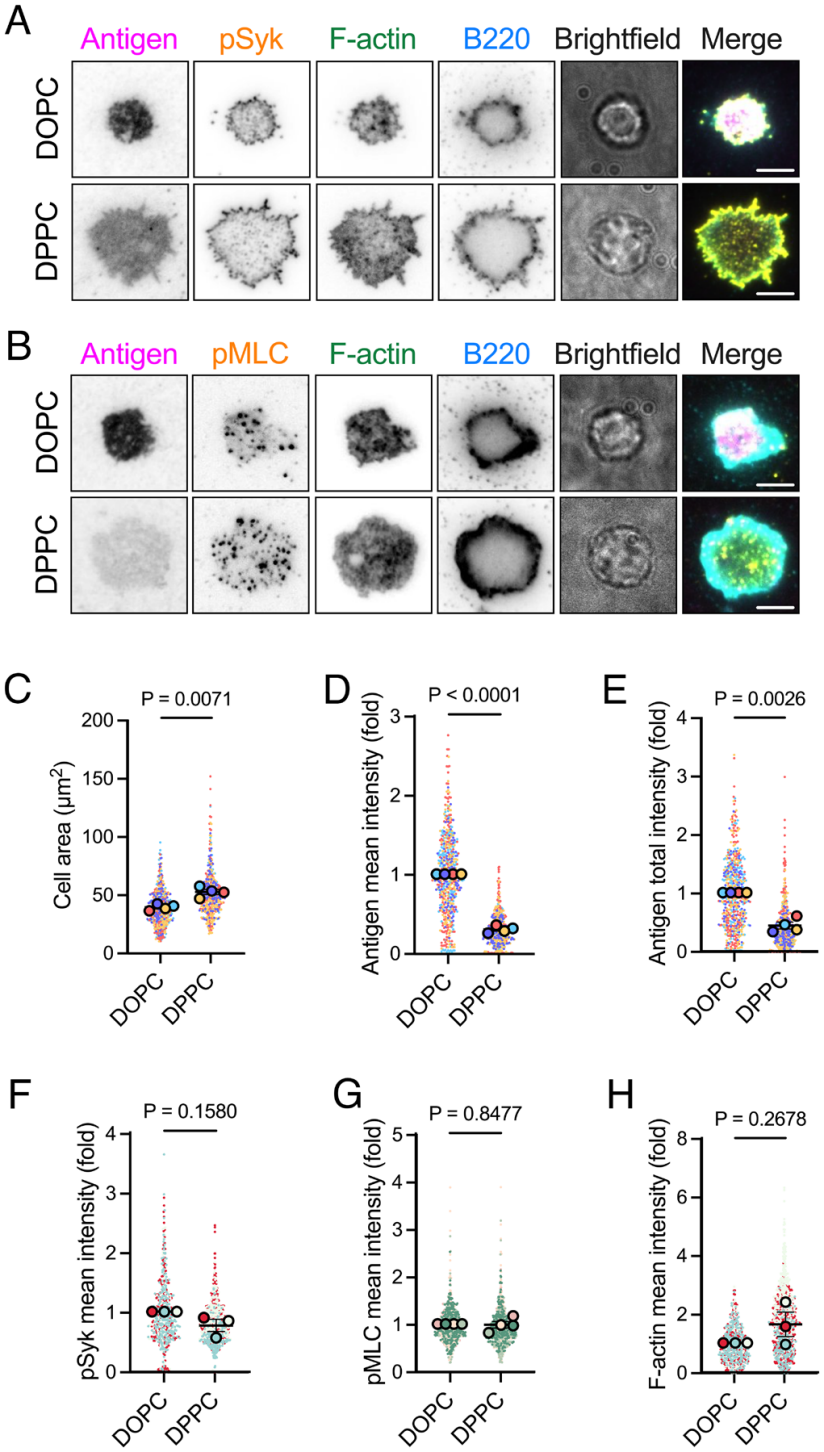
Lowering antigen mobility limits antigen binding but enhances B cell spreading and signaling. (*A* and *B*) TIRF microscopy images showing antigen, phosphoproteins, and filamentous (F)-actin distribution in B220+ B1-8 B cell synapses formed on DOPC (*Top*) and DPPC (*Bottom*) planar lipid bilayers (PLBs) after 15 min (*A*) or 10 min (*B*) stimulation with NIP_3_-DNA. (Scale bars, 5 µm.) (C-H) Quantification of synaptic parameters: cell spreading area (*C*), mean (*D*) and total (*E*) antigen accumulation measured at 45 min, and levels of phospho-Syk (pSyk) (15 min) (*F*), phospho-myosin light chain (pMLC) (10 min) (*G*), and F-actin (*H*) (15 min). Individual cells (solid dots) and experiment means (outlined dots) are shown, color-coded by experiment. Data represent three to four independent experiments with cell numbers: *C–E* (DOPC n = 533, DPPC n = 444, 4 experiments); *F* and *H* (DOPC n = 521, DPPC n = 888, 3 experiments); *G* (DOPC n = 636, DPPC n = 689, 4 experiments). Bars represent mean ± SEM. *P* values were determined by two-tailed paired *t* test comparing experiment means.

Using total internal reflection fluorescence (TIRF) microscopy and quantitative image analysis (*SI Appendix,* Fig. S4), we observed that cells spread more extensively on DPPC than on DOPC bilayers (Fig. 2 *A*–*C*), indicating that B cells exert higher traction forces on surfaces with greater viscosity (44). Though increased spreading has previously been associated with increased antigen binding (18), we observed that B cells formed smaller BCR–antigen microclusters and accumulated less antigen overall on DPPC compared to DOPC, as indicated by the mean (Fig. 2*D*) and total antigen intensity (Fig. 2*E*), respectively. On DOPC, B cells clustered large amounts of antigen at the center of the cell–bilayer contact, as typically observed on these fluid surfaces. In contrast, on DPPC, antigens remained distributed throughout the cell–bilayer interface and were less clustered.

Despite binding less antigen on DPPC, B cells phosphorylated Syk (Fig. 2*F*) and myosin light chain (Fig. 2*G*), and polymerized actin (Fig. 2*H*), as effectively as they did on DOPC. This suggests that BCR crosslinking by a trivalent antigen may be sufficient to induce robust signaling even in the absence of global BCR remodeling in the synapse (41), perhaps by bringing the local antigen concentration above a critical threshold needed for activation (52). Additionally, as the BCR is a mechanosensitive receptor (53), the higher traction forces facilitated by increased surface viscosity may amplify BCR signaling to compensate for reduced antigen binding.

### Calcium Flux and Transcription Factor Translocation Occur Effectively Across Both Substrate Viscosities.

The recruitment and phosphorylation of signaling proteins close to the BCR lead to the mobilization of intracellular calcium, which encodes information through its amplitude and oscillations (54). This, in turn, activates transcription factors such as Nuclear Factor Kappa B (NF-κB) and Nuclear Factor of Activated T cells (NFAT) to regulate long-term cellular processes including survival, proliferation, and differentiation (54, 55).

**Fig. 3.**
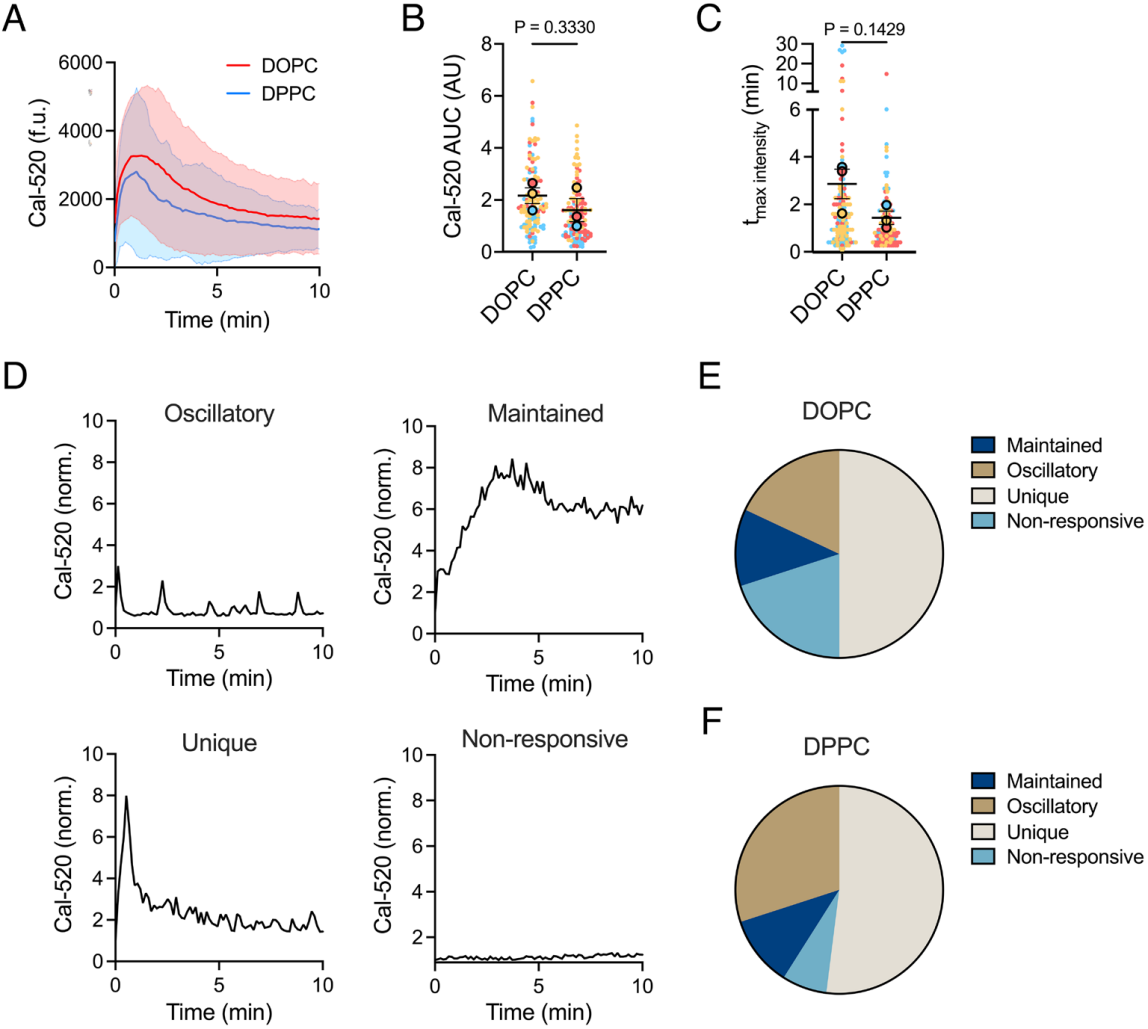
Antigen mobility does not impact B cell calcium signaling. (*A*–*F*) Analysis of calcium signaling in B1-8 naïve B cells responding to NIP_3_-DNA sensors. (*A*) Intracellular calcium levels over time on DOPC versus DPPC bilayers. Mean ± SD shown. (*B*) Total calcium response quantified as area under the curve (AUC, arbitrary units). (*C*) Time delay between initial BCR–antigen binding and peak calcium signal. (*D*) Representative calcium traces showing four distinct response patterns: oscillatory, maintained, unique, and nonresponsive. (*E* and *F*) Distribution of calcium response patterns on DOPC (*E*) and DPPC (*F*) bilayers. Data from 135 (DOPC) and 127 (DPPC) cells across three independent experiments. Individual cells (solid dots) and experiment means (outlined dots) are shown, color-coded by experiment. Bars in (*B* and *C*) represent mean ± SEM. *P* values were determined by two-tailed paired *t* test comparing experiment means.

We assessed intracellular calcium mobilization by loading B cells with the fluorogenic calcium-sensitive dye Cal-520 AM, seeding them onto NIP_3_-DNA-coated bilayers, and imaging them live at 37 °C. Imaging and quantification revealed that B cells responded to stimulation on DOPC with intracellular calcium fluxes slightly higher than those of B cells stimulated on DPPC (Fig. 3*A*), though the difference was not statistically significant (Fig. 3*B*). The calcium fluxes occurred with similar kinetics, as measured by the time delay between the cell first touching the bilayer and reaching maximum Cal-520 intensity (Fig. 3*C*).

**Fig. 4.**
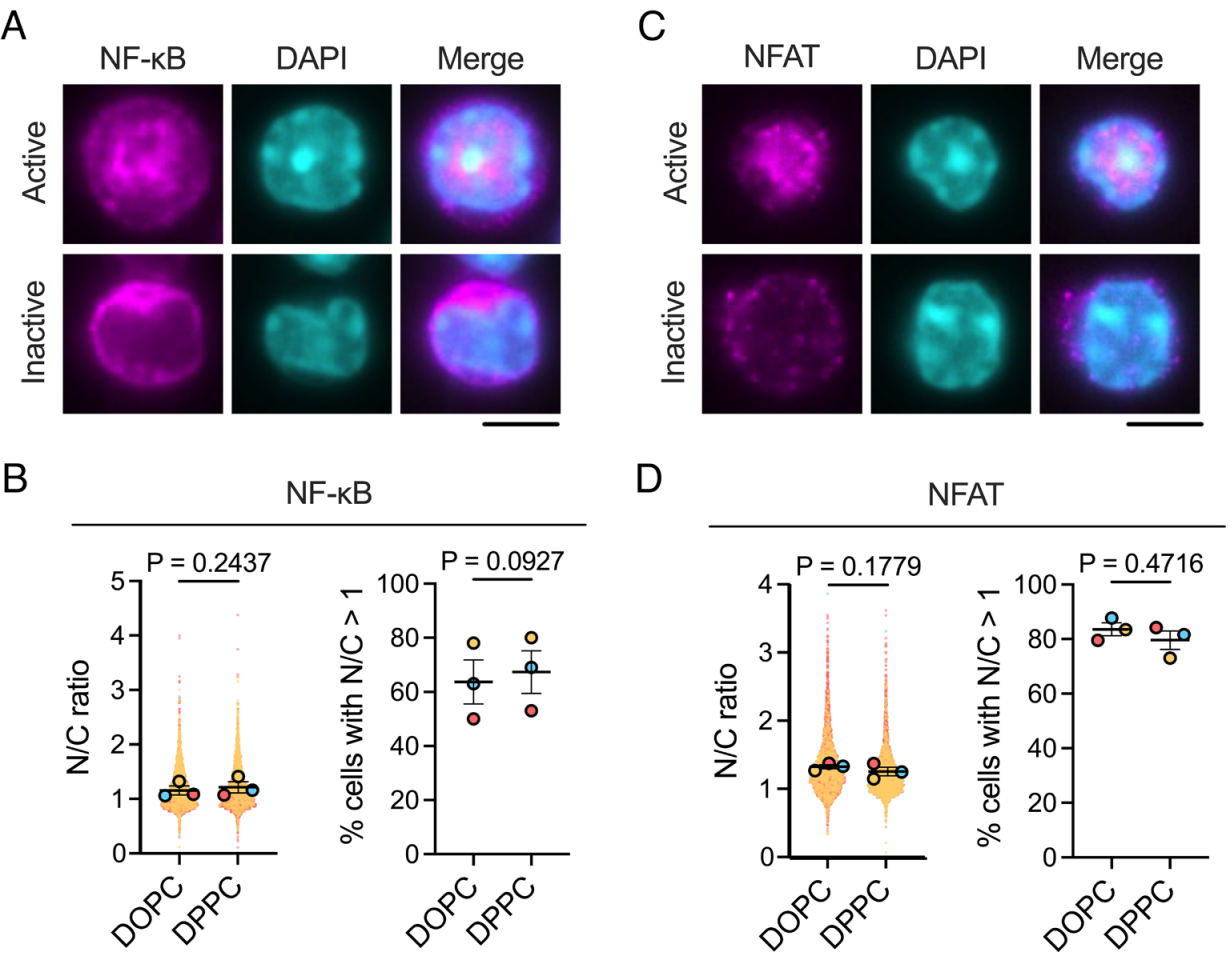
The activation of NF-κB and NFAT transcription factors is unaffected by antigen mobility. (*A*) Activation of NF-κB is measured on a cell-by-cell basis by calculating the nuclear-to-cytoplasmic ratio of transcription factor intensity. *Top*: active state with nuclear translocation; *Bottom*: inactive state with cytoplasmic retention. (Scale bar, 5 µm.) (*B*) Quantification of NF-κB activation, showing nuclear-to-cytoplasmic intensity ratios (I_nuc_/I_cyt_; *Left*) and percentage of activated cells (I_nuc_/I_cyt_ > 1; *Right*). (*C*) Activation of NFAT measured using the same nuclear-to-cytoplasmic ratio approach. (Scale bar, 5 µm.) (*D*) Quantification of NFAT activation, showing nuclear-to-cytoplasmic ratios (*Left*) and percentage of activated cells (*Right*). Data are from three independent experiments: NF-κB (DOPC n = 6,231; DPPC n = 4,221) and NFAT (DOPC n = 4,296; DPPC n = 3,498). Individual cells (solid dots) and experiment means (outlined dots) are shown, color-coded by experiment. Bars represent mean ± SEM. *P* values were determined by two-tailed paired *t* test comparing experiment means.

We also analyzed calcium oscillation patterns, categorizing the flux for each cell into one of four categories: oscillatory, maintained, unique, and nonresponsive (Fig. 3*D*). Cells were more likely to flux calcium on DPPC (93%) than on DOPC (80%). This is consistent with our hypothesis that antigens presented on higher viscosity membranes trigger BCRs more efficiently, aligning with our phospho-signaling observations (Fig. 2). Among responding cells, the distribution of calcium activation patterns remained similar between conditions, with the greatest fraction of cells having a single (unique) calcium peak (Fig. 3 *E* and *F*). Correspondingly, B cells stimulated by both surfaces translocated NF-κB component p65 (Fig. 4 *A* and *B*) and NFAT (Fig. 4 *C* and *D*) to the nucleus to the same extent. When defining activation as a nuclear-to-cytoplasmic fluorescence intensity ratio >1, we observed that approximately 66% of cells activated NF-κB (Fig. 4*B*) and 82% of cells activated NFAT (Fig. 4*D*). These results indicate that while antigen mobility influences the initial likelihood of a calcium response, both high- and low-mobility antigens elicit robust signal propagation through calcium release to NF-κB and NFAT, resulting in similar BCR-triggered signaling phenotypes.

### B Cells Extract Antigens More Efficiently From High-Viscosity Substrates.

The previous results suggested that substrate viscosity has little impact on BCR signaling in response to trivalent antigens. However, the reproductive fitness of a B cell during an immune response is primarily determined by the amount of antigen that B cells extract and internalize in the immune synapse. Therefore, we next focused on this aspect of B cell activation.

To quantify antigen extraction, we used a sensor containing a DNA duplex that can be unzipped to release NIP_3_ from the surface under an applied force of about 10 pN (*SI Appendix,* Fig. S5 and Table S3) (27). The sensor features an Atto647N fluorophore and Iowa Black RQ quencher pair at the base of the duplex. Upon duplex rupture, the Atto647N becomes fluorescent and is transported along with the NIP_3_ antigen into endosomal compartments. Since each NIP_3_ antigen is transported with one Atto647N fluorophore, the Atto647N fluorescence intensity serves as a proxy for the total amount of antigen captured by the cell. To quantify the total amount of antigen bound in the synapse, the bottom DNA strand—remaining on the substrate after duplex rupture—is labeled with an Atto550 fluorophore.

**Fig. 5.**
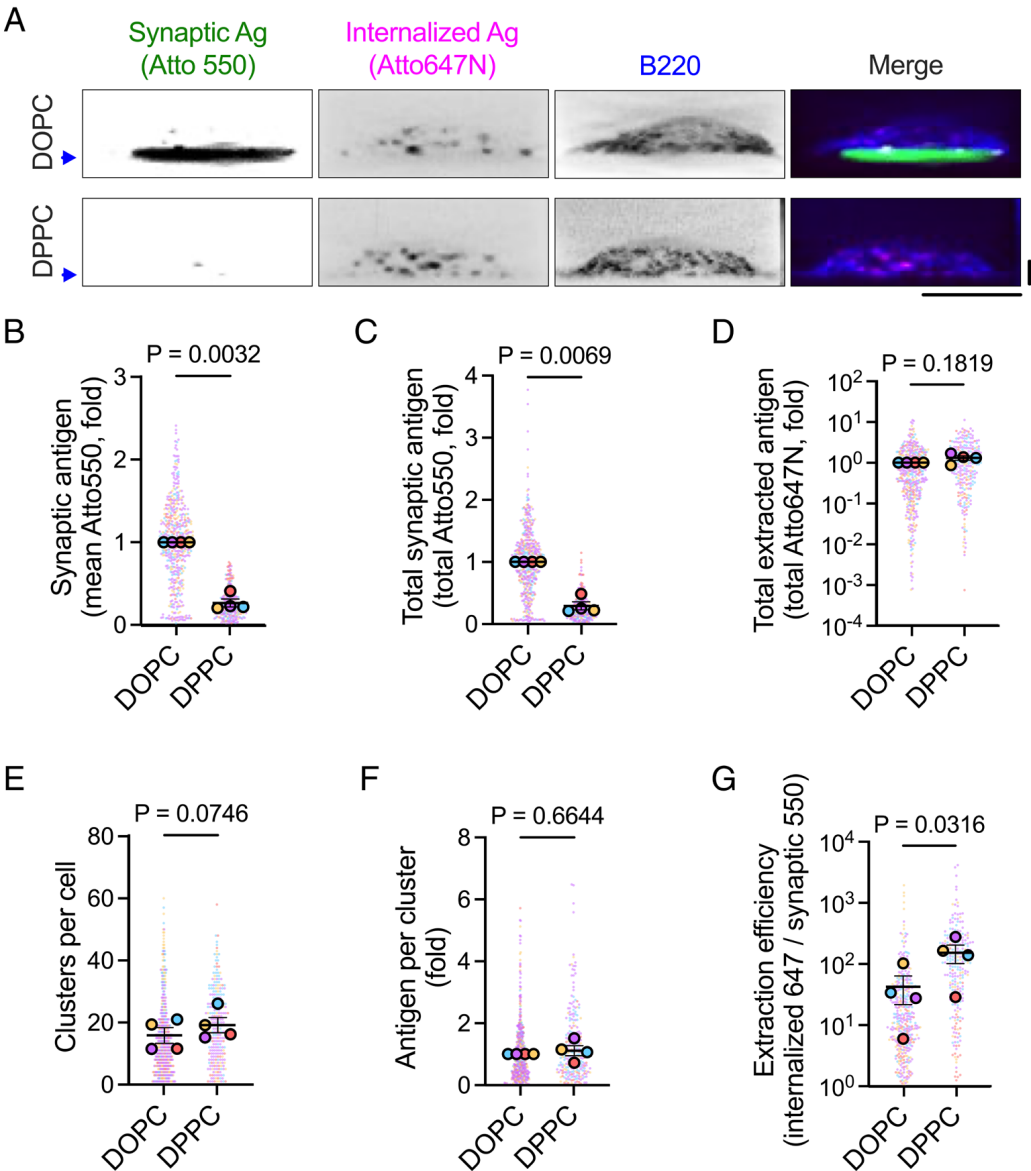
B cell antigen extraction is more efficient when antigens have low mobility. (*A*) Side-view reconstructions of B220+ B1-8 B cells showing membrane-bound (Atto550) and internalized (Atto647N) NIP_3_ antigen on DOPC and DPPC bilayers. Blue arrows indicate the bilayer positions. (Scale bars, 5 µm.) (*B* and *C*) Synaptic antigen accumulation after 45 min, quantified as mean (*B*) and total (*C*) intensity. (*D*–*F*) Characterization of extracted antigen clusters: total antigen extracted (*D*), clusters per cell (*E*), and antigen intensity per cluster (*F*). (*G*) Extraction efficiency was calculated as the ratio of internalized (Atto647N) to bound (Atto550) antigen intensity per cell. Data are from 472 (DOPC) and 264 (DPPC) cells across four independent experiments. Individual cells (solid dots) and experiment means (outlined dots) are shown, color-coded by experiment. Bars represent mean ± SEM. *P* values were determined by two-tailed paired *t* test comparing experiment means.

B cells accumulated less antigen in synapses formed on DPPC than on DOPC after 45 min of stimulation (Fig. 5*A*). This was assessed by quantifying the mean (Fig. 5*B*) and total (Fig. 5*C*) Atto550 signal intensity in the synapse and is consistent with earlier results using noninternalizing NIP_3_ antigens (Fig. 2 *D* and *E*). Despite binding substantially less antigen on DPPC bilayers, B cells internalized comparable amounts relative to cells on DOPC, as evidenced by the total antigen extracted (Fig. 5*D*), the number of extracted antigen clusters (Fig. 5*E*), and the antigen quantity per cluster (Fig. 5*F*) (all evaluated in the Atto647N channel). Consequently, B cells internalize a larger proportion of available antigen molecules in synapses formed on DPPC bilayers (Fig. 5*G*), indicating that high-viscosity substrates enhance the efficiency of antigen capture by B cells.

### Substrate Viscosity Influences the Spatiotemporal Dynamics of Antigen Extraction in the Synapse.

**Fig. 6.**
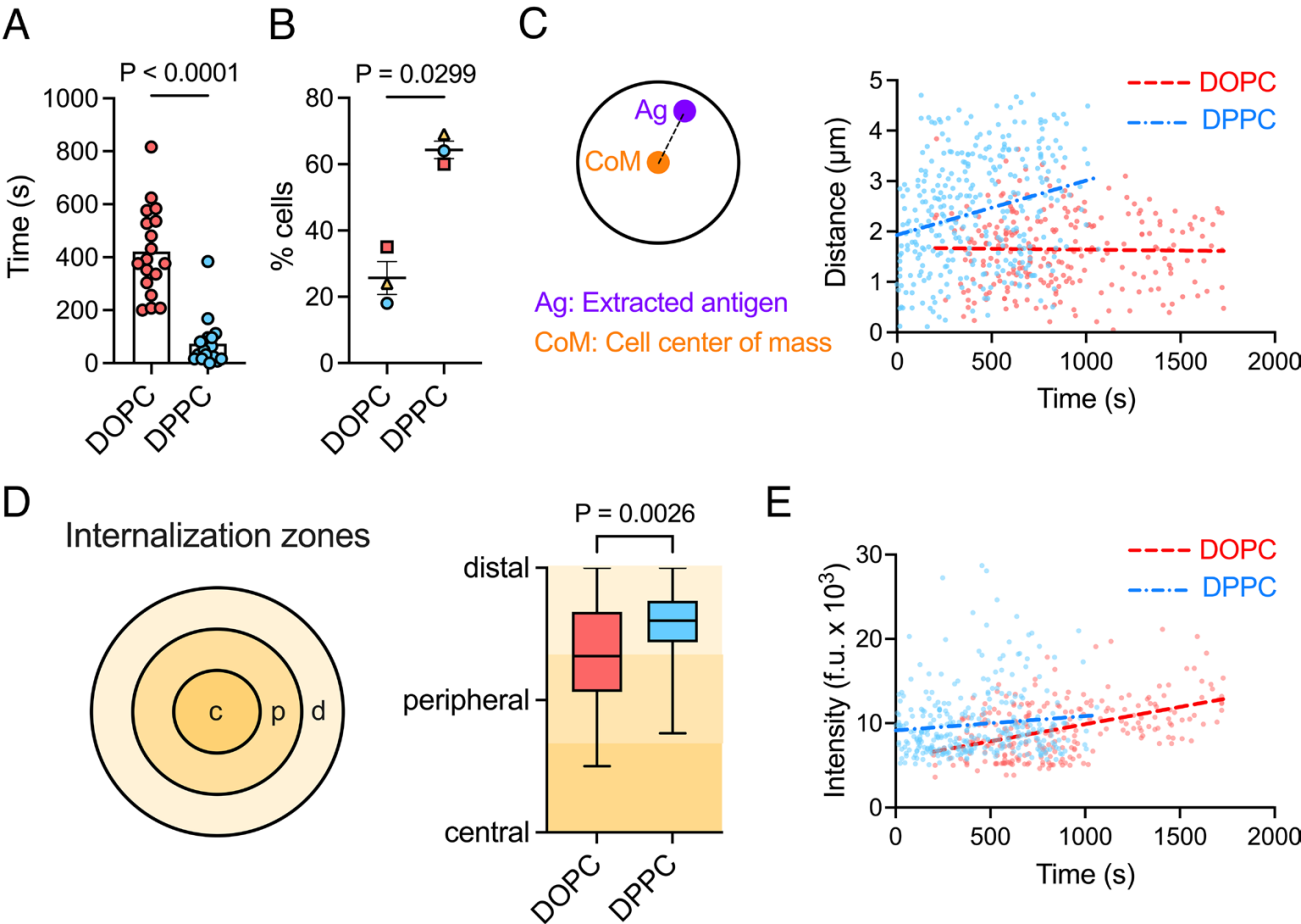
B cells extract antigens with different spatiotemporal dynamics from DOPC and DPPC bilayers. (*A*) Time to first antigen cluster extraction after initial bilayer contact. Individual cells are shown. (*B*) Percentage of cells extracting at least one antigen cluster within 5 min of contacting the bilayer. The points show experiment means and bars represent mean ± SEM. (*C*) Relationship between extraction time and distance from the cell center. Pearson correlation analysis shows a positive correlation on DPPC (r = 0.3, *P* < 0.0001) but not on DOPC (r = −0.02, *P* = 0.77). (*D*) Distribution of extraction events across central, peripheral, and distal synaptic regions. Boxes indicate the quartile range (first quartile to third quartile), the horizontal line indicates the median value, and the whiskers extend to the minimum and maximum points. (*E*) Cluster size (Atto647N intensity) versus extraction time. Pearson correlation analysis shows a positive correlation on both substrates: DOPC (r = 0.47, *P* < 0.0001), DPPC (r = 0.11, *P* = 0.0457). Data represent 245 (DOPC) and 324 (DPPC) antigen clusters extracted by 18 cells per condition, pooled from three independent experiments. Each dot in C and E represents a single extracted cluster. *P* values in *A* and *B* were determined by two-tailed paired *t* test comparing experiment means.

While measuring antigen extraction after 45 min reveals B cells’ capacity to acquire antigens from presenting membranes, it does not capture the dynamics of this process. Given the transient nature of B cell contacts with APCs (12, 15), we sought to determine whether differences in antigen mobility affected the kinetics of antigen capture. This was possible because mechanical rupture of the DNA duplex leads to Atto647N unquenching and the appearance of distinct fluorescent spots that can be detected and tracked by live-cell microscopy (*SI Appendix,* Fig. S6 and Movie S3). By quantifying the time between a cell first contacting the bilayer and internalizing its first antigen cluster, we found that B cells internalized antigen significantly faster from DPPC (mean: 74 s) compared to DOPC (mean: 421 s) (Fig. 6*A*). Further, 64% of B cells internalized at least one antigen cluster from DPPC within 5 min, compared to only 26% from DOPC (Fig. 6*B*). These results indicate that lowering antigen mobility enables B cells to capture antigens on the fast timescales of B cell–APC interactions in vivo.

A current hypothesis suggests that the centralization of antigens within the synapse is necessary for their extraction and internalization by B cells (56). To test this, we analyzed the spatial coordinates of extracted antigen clusters from single-particle trajectories, measuring the distance of each cluster from the cell center using an anti-IgM Fab surface label to define the cell boundary (Fig. 6 *C*, *Left*). A plot of distance versus time revealed distinct spatiotemporal dynamics of antigen extraction from DOPC and DPPC bilayers (Fig. 6 *C*, *Right*). On DPPC, B cells initially internalized antigens at a mean distance of 1.9 µm from the cell center, increasing to 3 µm by 17 min. In contrast, on DOPC, internalization consistently occurred at a mean distance of 1.7 µm from the cell center over 30 min. This suggests that antigen mobility influences the location of extraction within the synapse.

Given that B cells spread differently on DOPC and DPPC bilayers, we further analyzed the data by dividing the synapse into three concentric regions of equal width—central, peripheral, and distal zones—and quantifying the fraction of antigen clusters extracted from each region (Fig. 6 *D*, *Left*). These zones were recalculated for each frame in the time-lapse videos to account for changing cell area over time. The results showed that antigen extraction is skewed toward the cell edge, with >80% of clusters on DOPC and >90% of clusters on DPPC extracted from either the peripheral or distal regions (Fig. 6 *D*, *Right*). Only 15% on DOPC and 7% on DPPC were extracted from the synapse center. Thus, centralization is not required for B cells to capture antigens from presenting membranes.

B cells cluster antigens to different extents in synapses formed on DOPC and DPPC bilayers (Fig. 2 *D* and *E*). We therefore investigated whether the size of extracted antigen clusters changes over time to reflect variations in antigen accumulation in the synapse. We quantified the intensity of each extracted antigen cluster in its initial detection frame and plotted intensities against extraction time (Fig. 6*E*). A positive correlation was found on both DOPC (r = 0.47, *P* < 0.0001) and DPPC (r = 0.11, *P* = 0.0457), indicating that antigen clustering in the synapse promotes the extraction of larger antigen clusters. This effect was more pronounced on DOPC, reflecting the greater extent of antigen clustering on this highly mobile substrate.

### Internalized Antigens are Transported to MHCII+ Compartments Consistently Across Varying Substrate Viscosities.

**Fig. 7.**
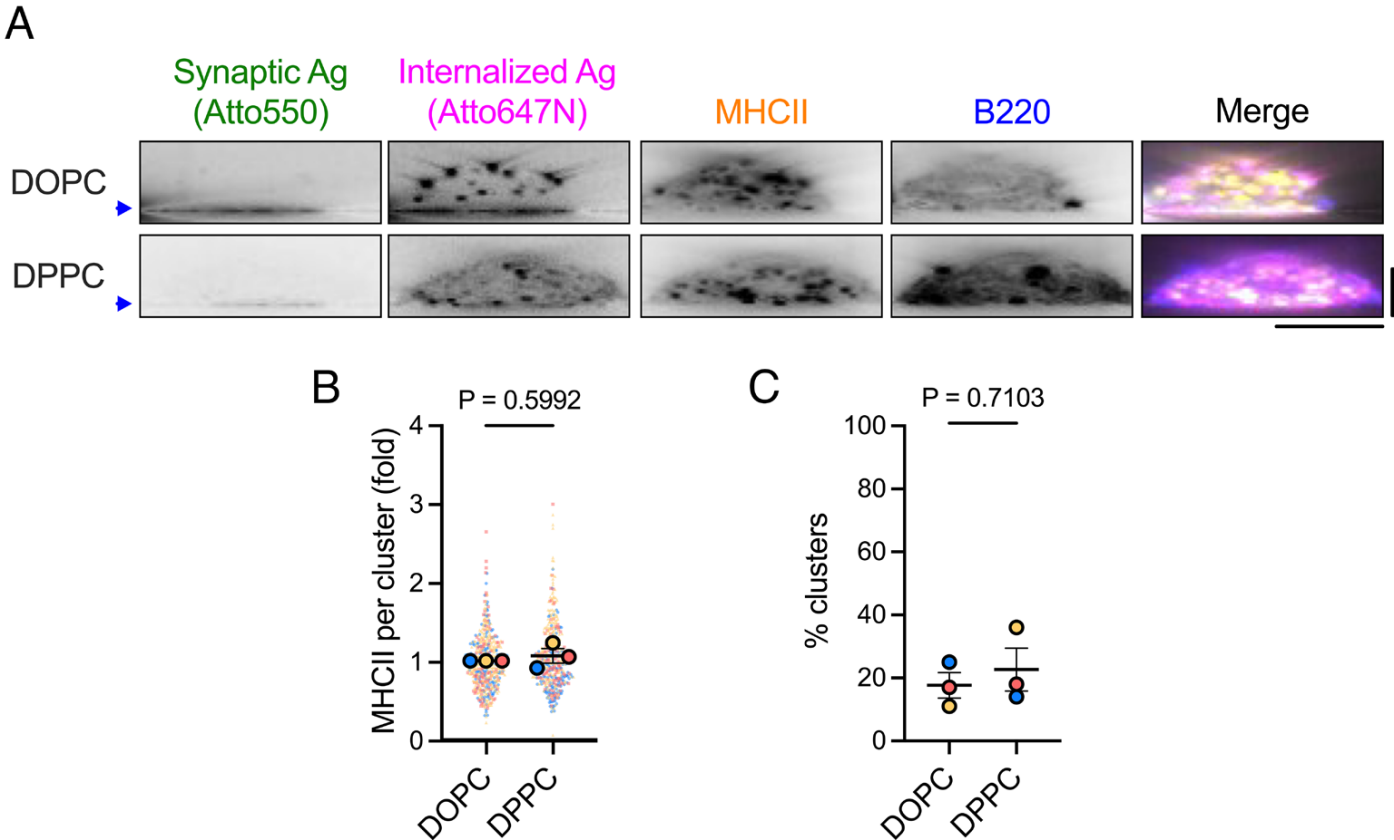
B cells traffic antigens extracted from DOPC and DPPC membranes to MHCII+ compartments at the same rate. (*A*) Side-view reconstructions showing B220+ cells with internalized NIP_3_ antigen clusters colocalizing with MHCII. The blue arrows indicate bilayer positions. [Scale bars, 5 µm (horizontal) and 10 µm (vertical).] (*B* and *C*) Quantification of MHCII+ trafficking: the mean MHCII intensity per internalized cluster (*B*) and the percentage of MHCII+ clusters (*C*). Data are from 538 (DOPC) and 475 (DPPC) cells across three independent experiments. Individual cells (solid dots) and experiment means (outlined dots) are shown, color-coded by experiment. Bars represent mean ± SEM. *P* values were determined by two-tailed paired *t* test comparing experiment means.

It is crucial for the antibody response that antigens are delivered to MHCII+ compartments for processing and presentation. The observation that B cells capture antigens from DOPC and DPPC bilayers with distinct spatial and temporal kinetics led us to question whether the antigens may be trafficked differently within the cell. To investigate this, we allowed B cells to interact with NIP_3_-DNA-coated bilayers for 20 min and then fixed and stained them intracellularly with an anti-MHCII antibody. Z-stack images and quantification revealed that internalized antigen clusters were trafficked to MHCII+ compartments (Fig. 7*A*), as assessed both by the intensity of anti-MHCII staining per internalized antigen cluster (Fig. 7*B*) and the percentage of internalized antigen clusters that colocalized with MHCII (Fig. 7*C*). These findings suggest that despite differences in the efficiency, dynamics, and location of antigen internalization, B cells direct internalized antigens to the correct intracellular compartments for processing and presentation.

### High Substrate Viscosity Enhances B Cell Discrimination of Antigen Affinities.

**Fig. 8.**
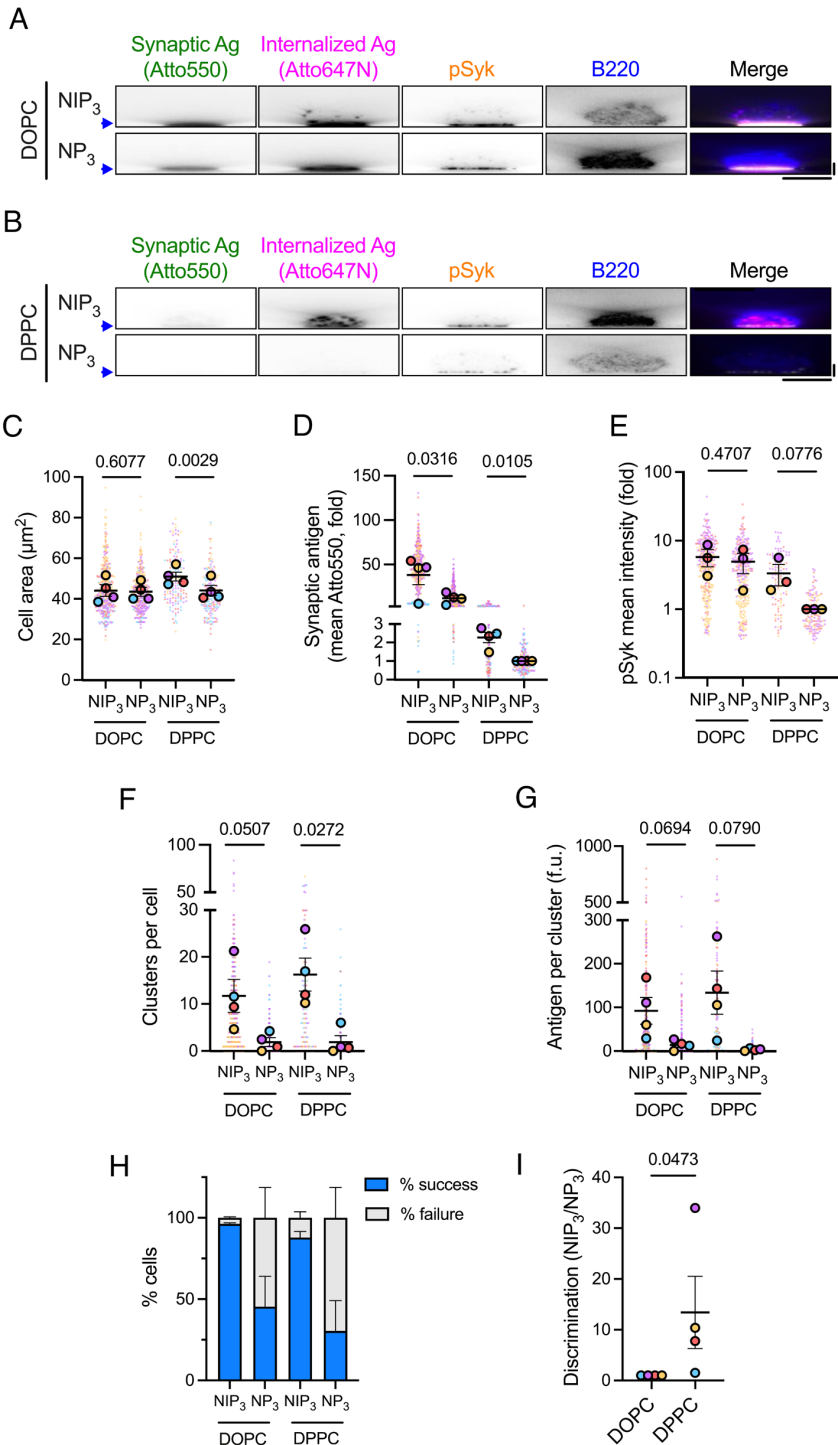
B cell antigen extraction is more stringent from high-viscosity substrates. (*A* and *B*) Side-view reconstructions of B220+ B1-8 B cells showing membrane-bound (Atto550) and internalized (Atto647N) NIP_3_ antigen and phospho-Syk signaling on DOPC (*A*) and DPPC (*B*) bilayers. Blue arrows indicate the bilayer positions. (Scale bars, 5 µm.) (C–E) Quantification of cell spreading area (*C*), antigen clustering (*D*), and phospho-Syk signaling (*E*) in synapses formed on DOPC versus DPPC bilayers. (*F*–*H*) Effect of substrate viscosity on the number of internalized clusters (*F*), the amount of antigen in each internalized cluster (*G*), and the percentage of cells internalizing at least one cluster (*H*). (*I*) The ratio of NIP_3_ to NP_3_ antigen internalized from DOPC and DPPC. Data are from four independent experiments: DOPC (NIP_3_: 410 cells, NP_3_: 368 cells) and DPPC (NIP_3_: 145 cells, NP_3_: 185 cells). pSyk (E) was measured in three of the four experiments. Individual cells (solid dots) and experiment means (outlined dots) are shown, color-coded by experiment. Bars represent mean ± SEM. *P* values were determined by two-tailed paired *t* test comparing experiment means.

We previously demonstrated that substrate stiffness affects the forces applied to BCR–antigen bonds, influencing B cells’ ability to discriminate antigen affinities (27). To determine whether antigen mobility has a similar effect, we compared the ability of B cells to distinguish NIP_3_ from NP_3_ (4-hydroxy-3-nitrophenyl), a hapten that binds the B1-8 Fab with ~10-fold lower affinity (21) (Fig. 8 *A* and *B*). After 45 min, B cells had discriminated NIP_3_ and NP_3_ on DPPC, as shown by cell spread area (Fig. 8*C*), antigen binding (Fig. 8*D*), and Syk phosphorylation (Fig. 8*E*). On DOPC, discrimination was more modest, primarily reflected as a difference in antigen binding (Fig. 8*D*). The strongest effects were observed in antigen extraction, where B cells internalized significantly more NIP_3_ than NP_3_ from both substrates, measured by the number of internalized clusters per cell (Fig. 8*F*), the amount of antigen in each internalized cluster (Fig. 8*G*), and the percentage of cells acquiring at least one antigen cluster (Fig. 8*H*). However, the NIP_3_ to NP_3_ extraction ratio was significantly higher on DPPC (Fig. 8*I*), indicating that B cells achieve more stringent affinity discrimination when antigens have low mobility.

## Discussion

B cells use mechanical energy to physically extract antigens from the surfaces of other cells (21). This process serves two purposes: internalizing antigens for processing and presentation (24), and discriminating antigens based on the force-dependent lifetimes of BCR–antigen bonds (57). Our study reveals that antigen mobility regulates the magnitude, location, and timing of forces in the immune synapse, thereby regulating the speed and accuracy of B cell antigen capture and discrimination.

The actin cytoskeleton plays a central role in B cell activation, orchestrating immune synapse formation through rapid self-remodeling and consequent cell surface restructuring in response to BCR signaling. Actin polymerization at the leading cell edge drives cell spreading (18), while membrane tension together with myosin IIa contractility generate a retrograde actin flow that facilitates BCR–antigen clustering, signaling, and centripetal transport (58). Our study confirms previous observations that antigen mobility influences these actin-dependent behaviors, with highly mobile antigens enhancing clustering and transport, and low-mobility antigens promoting cell spreading (33). However, unlike earlier studies using monovalent antigens (30, 33), we observed no enhancement in proximal BCR signaling with increased antigen mobility. This extended to downstream processes, including calcium flux and the activation of NFAT and NF-κB transcription factors. These findings align with our recent work characterizing B cell activation by subcapsular sinus macrophages (32), suggesting that antigen mobility does not alter B cell fitness. The discrepancy with earlier work likely stems from our use of antigen trimers, which can crosslink multiple BCRs to initiate robust signaling (41). Antigen trimers feature in various pathogens, including viral spike proteins (e.g., HIV-1, SARS-CoV-2, influenza hemagglutinin) (59–61) and bacterial proteins (e.g., OmpF, TAAs) (62, 63), potentially representing an optimal antigenic unit for eliciting robust B cell activation across diverse physical environments.

Actin remodeling and myosin II contractility not only reshape the immune synapse but also exert mechanical forces on BCR–antigen bonds. Using antigen extraction as a proxy for force generation, we demonstrate that these synaptic forces exhibit distinct dynamics depending on antigen mobility. B cells begin extracting antigen from DPPC membranes rapidly, within 8 s of contact, while extraction from DOPC membranes begins after 200 s. Despite this temporal difference, cells ultimately capture similar amounts of antigen from both substrates by 45 min. This occurs because cells on DPPC extract antigens efficiently but only from their initial contact area, whereas cells on DOPC can continuously recruit and extract antigens from the surrounding bilayer, accessing two- to threefold more antigen overall. Thus, while extraction is less efficient on DOPC, the larger pool of accessible antigen enables cells to eventually achieve extraction levels comparable to those on DPPC.

The spatial distribution of extraction also differs between substrates. Antigens presented on fluid-phase DOPC membranes are most likely extracted in the peripheral synapse region, where myosin II motors contract actin filaments to generate traction forces (20, 34, 35). In contrast, antigens displayed on gel-phase DPPC membranes are mainly extracted in the distal synapse region, where forces arise from rapid retrograde actin flow (20, 64). While we observe some antigen extraction in the central area of the synapse, consistent with previous studies (35, 36), this accounts for only a minor fraction of total antigen uptake. This suggests that flowing actin filaments, rather than dynamic actin puncta, predominate during antigen capture in the B cell synapse.

The coupling between actin flow and cellular adhesion complexes is a well-described mechanism for force transmission from a cell to its substrate (38, 65). These forces are, in turn, influenced by the substrate’s mechanical properties (44, 66, 67). When B cells pull on antigens embedded in a fluid membrane, much of the energy dissipates due to antigen and substrate remodeling that limits the shear forces that B cells can apply through retrograde actin flow. As a result, B cells primarily rely on tensile forces exerted by myosin II motors in the synapse periphery. Conversely, low-mobility antigens largely retain their structure within gel-phase membranes, directing more mechanical energy to BCR–antigen bonds and allowing B cells to leverage the high actin flow velocities in the distal region of the synapse for antigen extraction. Restricted growth of antigen clusters on gel-phase substrates also increases the mechanical load on individual BCR–antigen bonds (22, 68, 69). Higher forces not only increase the probability of antigen extraction through enhanced rupture of soft DNA tethers (26), but also shorten and narrow bond lifetime distributions (70), leading to improved antigen affinity discrimination and faster antigen capture. These results suggest that antigens of different mobility engage distinct cytoskeletal structures within the immune synapse, altering the fidelity of B cell selection.

That only ~20% of internalized antigens are observed within MHCII+ compartments in our experiments highlights a need to further investigate how antigen mobility and synaptic patterning influence downstream B cell outcomes. It is possible that our fixed time-course imaging measurements capture only a fraction of the total antigens in these compartments as they traffic through complex and time-dependent routes required for processing and MHCII presentation (71). An alternative explanation is that B cells internalize antigens through different pathways depending on substrate mobility. B cells use at least two endocytic routes—clathrin-mediated endocytosis (21) and fast endophilin-mediated endocytosis (72)—for internalizing membrane-presented antigens. The proportion of antigens directed toward each pathway could be influenced by antigen cluster size (73) or mechanical tension (74), both of which we demonstrate are affected by antigen mobility. This could have important implications for B cell fate, as these distinct internalization routes not only provide metabolic support for B cell growth and proliferation (72), but also shape the peptide repertoire presented on MHCII (71). Further investigation into how antigen mobility affects antigen trafficking and presentation could provide valuable insights into B cell activation and immune function.

Our hybrid live cell-supported membrane platform has revealed that antigen mobility directly influences the speed and accuracy of naïve B cell activation. This finding is physiologically relevant, as APCs use their actin cytoskeleton to restrict antigen diffusion and cluster growth (32), potentially enhancing the activation of high-affinity B cells over lower affinity cells by maximizing forces on BCR–antigen bonds. However, the situation is complex. B cell antigen discrimination is also sensitive to APC membrane stiffness (27), which varies across cell types and is modulated by inflammatory signals (75). APCs can dynamically adapt their physical properties in response to changing mechanics and biochemical composition of the extracellular matrix (32), which is remodeled during the adaptive immune response (76, 77).

The complexity extends beyond APC mechanical properties. APCs control both the mobility and densities of antigens on their surfaces (32, 49), influencing the strength of B cell activation (18). Future studies will need to explore how antigen mobility and density work together to regulate B cell antigen capture and discrimination. This may be particularly important at low antigen densities, where increased mobility could potentially help B cells accumulate sufficient antigens to reach activation thresholds. Furthermore, B cells undergo significant changes during immune responses, differentiating into germinal center and memory B cells with class-switched BCRs and distinct cytoskeletal architectures that alter mechanotransduction (22, 78, 79). This suggests that factors beyond BCR affinity are likely to influence the antibody repertoire, potentially helping to explain recent findings that germinal centers support the maturation of B cells with a breadth of affinities to yield diverse antibody responses (80, 81).

## Materials and Methods

### Antigen-Conjugated DNA Probes.

The NIP_3_-conjugated DNA constructs were prepared and characterized as described in *SI Appendix*, *Materials and Methods*. DNA sequences are provided in *SI Appendix*, Tables S2 and S3.

### Primary B Cell Harvesting and Culture.

All animal work was performed with prior approval from the UK Home Office and the King’s College London Ethical Review Panel. Naïve B cells were harvested from the spleens of B1-8^flox/flox^ Igκ^Ctm1Cgn/tm1Cgn^ mice and cultured in full RPMI (RPMI 1640 medium supplemented with 10% FBS, 1% MEM nonessential amino acids, 2 mM L-glutamine, 50 µM 2-mercaptoethanol, 100 U/ml penicillin, and 100 µg/ml streptomycin) at 37 °C with 5% CO_2_ for 1 to 2 h before using in experiments. Full details on B cell harvesting and culture are provided in *SI Appendix*, *Materials and Methods*.

### PLB Preparation.

PLBs were composed of either 97 mol% 1,2-dioleoyl-sn-glycero-3-phosphocholine (DOPC) or 97 mol% 1,2-dipalmitoyl-sn-glycero-3-phosphocholine (DPPC), 2 mol% 1,2-dipalmitoyl-sn-glycero-3-[(N-(5-amino-1-carboxypentyl)iminodiacetic acid)succinyl] (nickel salt) [DGS-NTA(Ni)], and 1 mol% 1,2-dioleoyl-sn-glycero-3-phosphoethanolamine-N-(cap biotinyl) (sodium salt) (biotin-DOPE). Full details for bilayer preparation, conjugation with NIP_3_-conjugated DNA constructs, and measurement of NIP_3_-DNA surface density are given in *SI Appendix*, *Materials and Methods*.

### Microscopy.

A Nikon Ti Eclipse motorized inverted microscope was used to collect all cell images as described in *SI Appendix*, *Materials and Methods*. Microscope hardware was controlled for image acquisition using Micromanager software (82).

TIRF images were acquired for analyzing phospho-signaling and F-actin accumulation in the immune synapse, calcium signaling, and antigen-DNA diffusion, as described in *SI Appendix*, *Materials and Methods*. Widefield z-stack images were acquired to quantify fixed-cell antigen internalization and transcription factor translocation as described in refs. 22, 32 and in *SI Appendix*, *Materials and Methods*. Live-cell measurements of antigen extraction were acquired in widefield, focused on the bilayer plane.

### Reagents for Fixed-Cell Imaging.

The antibodies and probes used for fluorescence staining are provided in *SI Appendix*, Table S4.

### Figures and Statistics.

Graphs were plotted using SuperPlots formatting (83), where data for individual cells (small data points) and the mean values per experiment (large, outlined data points) were plotted together. Graphing and statistical analysis were performed using GraphPad Prism (versions 9 and 10). Where possible, exact *P* values are shown on the plots. *P* > 0.05 is considered nonsignificant (ns) and *P* ≤ 0.05 is considered significant (*P ≤ 0.05, **P ≤ 0.01, ***P ≤ 0.001, and ****P ≤ 0.0001). A description of statistical tests and number of independent experiments is provided in the figure legends.

## Supplementary Material

Appendix 01 (PDF)

Movie S1. **Single-particle imaging of antigen-DNA complexes on DOPC**. Images were acquired with 10 ms exposure time and 0 s between frames.

Movie S2. **Single-particle imaging of antigen-DNA complexes on DPPC**. Images were acquired with 10 ms exposure time and 0 s between frames.

Movie S3. **Live-cell imaging of antigen extraction from bilayer substrates**. Dark spots appear when B cells rupture antigens from the DNA tether, separating an Atto647N fluorophore from a dark quencher. Extraction from DOPC is shown on the left, and from DPPC on the right. Images were acquired every 8 s. Scale bar: 5 μm.

## Data Availability

Analysed data sets have been deposited in the Imperial College London Research Data Repository (https://doi.org/10.14469/hpc/15026) (84). The ImageJ/Fiji, CellProfiler, Icy, and Python scripts used for image analysis are available on GitHub (https://github.com/SpillaneLab) (85). All other data are included in the article and/or supporting information.
