# Supplementary material for: Antigen mobility regulates the dynamics and precision of antigen capture in the B cell immune synapse
Source: Proc Natl Acad Sci U S A. 2025 May 12;122(20):e2422528122. doi: 10.1073/pnas.2422528122 (PMC12107191; doi:10.1073/pnas.2422528122)
Supplement: Supplementary file 1 — Appendix 01 (PDF) [file pnas.2422528122.sapp.pdf]

PNAS

Supporting Information for

Antigen mobility regulates the dynamics and precision of antigen capture in the B cell immune synapse

Hannah C. W. McArthur, Anna T. Bajur, Maro Iliopoulou, and Katelyn M. Spillane

Correspondence: Katelyn M. Spillane
Email: k.spillane@imperial.ac.uk

This PDF file includes:

SI Materials and Methods
Figures S1 to S6
Tables S1 to S4
Legends for Movies S1 to S3
SI References

Other supporting materials for this manuscript include the following:

Movies S1 to S3

SI Materials and Methods

Mice. B1-8$^{flox/flox}$ Igκ$^{Ctm1Cgn/tm1Cgn}$ mice on a C57BL/6 background (B1-8 mice) were used as a source of primary naïve B cells for all experiments. All mice were between 14 and 22 weeks old and both males and females were used. Mice were bred and treated in accordance with guidelines set by the UK Home Office and the King's College London Ethical Review Panel.

Primary B cell isolation and culture. For each experiment, primary splenocytes were obtained by passing a mouse spleen through a 70-μm cell strainer and lysing red blood cells with RBC lysis buffer (Invitrogen). Cells were pelleted by centrifugation for 7 minutes at 300x*g* and 4 °C, resuspended in 450 μl ice-cold MACS buffer (PBS, pH 7.3, 0.5% BSA, 1 mM EDTA), and incubated with 50 μl CD43 (Ly-48) MicroBeads, mouse (Miltenyi Biotec) for 20 minutes on ice. The cells were resuspended in 10 ml ice-cold MACS buffer, pelleted, resuspended in 500 μl ice-code MACS buffer, and isolated by negative selection using an LD column and MidiMACS Separator (Miltenyi Biotec). Cells were pelleted and resuspended to a density of 5 x 10^6 cell/ml in full RPMI (RPMI 1640 medium supplemented with 10% FBS, 1% MEM non-essential amino acids, 2 mM L-glutamine, 50 μM 2-mercaptoethanol, 100 U/ml penicillin, and 100 μg/ml streptomycin, all from Gibco), and cultured at 37 °C with 5% CO_2 for 1-2 h before using in experiments.

Preparation of glass coverslips. Glass coverslips (24 x 50 mm, No. 1.5; Fisher) were etched in piranha solution (2:1 H_2SO_4:30% H_2O_2) for 15 minutes, washed 10 times with ultrapure water (Sartorius Arium), and rinsed three times with 100% ethanol. Sample chambers were assembled by placing a strip of 10 μl CultureWell gaskets (Grace Bio-Labs) onto the coverslip, which was then glued onto a one-well Nunc Lab-Tek chamber (Thermo Fisher Scientific) using Sylgard fast-cure silicone (Silmid).

Antigen-conjugated DNA tension sensors. The sensors were assembled as previously described (1). Briefly, HPLC-purified single-stranded DNA oligos (Tables S2 and S3) were purchased from Integrated DNA technologies and resuspended to 100 μM in duplex buffer (30 mM HEPES, pH 7.5, 100 mM potassium acetate; IDT). To assemble the sensors, oligos were mixed in stoichiometric amounts, supplemented with 2 mM $MgCl_2$, heated to 95 °C for 3 minutes in a thermocycler, and cooled on the bench top to room temperature for 30 minutes. The annealed sensor was then exchanged into degassed 0.1 M sodium carbonate buffer, pH 8.3, using a 7-kDa molecular weight cut-off (MWCO) desalting column (Zeba; Thermo Fisher Scientific) and incubated with a 20-fold molar excess of either NIP-Osu (4-hydroxy-3-iodo-5-nitrophenylacetic active ester; Biosearch Technologies) or NP-Osu (4-hydroxy-3-nitrophenyl hapten active ester; Biosearch Technologies) per Uni-link modifier for 1 hour at room temperature to label the amine functional

groups. The solution was then passed twice through desalting columns to remove unreacted hapten. A conjugation ratio of 3:1 was confirmed by UV-Visible spectroscopy (NanoDrop One^{C}; Thermo Fisher Scientific) using the Atto550 (rupturable sensor) or Atto647N (non-rupturable sensor) absorbance as a proxy for DNA concentration. The 3:1 hapten:DNA ratio measured by UV-Visible spectroscopy, combined with the presence of three Uni-Link modifications as the only available coupling sites on each DNA molecule, provides evidence for complete trivalent modification rather than a statistical distribution of partially modified products. The sensors were stored at –20 °C in single-use aliquots.

Estimating inter-hapten distance. Computational analysis of the trivalent antigen followed a two-step approach combining Avogadro and CREST calculations. The initial fully extended structure was generated in Avogadro (2) to estimate the maximum inter-hapten distance. To determine minimum inter-hapten distance, we first optimized the structure using xTB-6.6.1, using the semiempirical tight binding-based quantum chemistry method GFN-xTB with implicit water solvation (3). We then used CREST-2.12 to identify the lowest energy conformer through computational sampling (4). The simulation was run for 0.5 ps using the GFN2-xTB method with implicit water solvation, maintaining all other parameters at default values. Molecular structures from these analyses (Fig. S1) were visualized using CYLview20 (C. Y. Legault, Université de Sherbrooke, http://www.cylview.org), with inter-hapten distances measured in Avogadro.

Planar lipid bilayers. Small unilamellar vesicles (SUVs) were prepared by mixing either 97% 1,2-dioleoyl-sn-glycero-3-phosphocholine (DOPC) or 97% 1,2-dipalmitoyl-sn-glycero-3-phosphocholine (DPPC), 2% 1,2-dipalmitoyl-sn-glycero-3-[(N-(5-amino-1-carboxypentyl)iminodiacetic acid)succinyl] (nickel salt) (DGS-NTA(Ni)), and 1% 1,2-dioleoyl-sn-glycero-3-phosphoethanolamine-N-(cap biotinyl) (sodium salt) (biotin-DOPE; Avanti Polar Lipids, Inc.) in chloroform at a final lipid concentration of 4 mg/ml (5). The solvent was dried with a gentle stream of argon and then under vacuum for at least 3 h. The lipid film was resuspended to 5 mM in degassed PBS at room temperature (DOPC) or degassed SUV buffer (DPPC; 10 mM Tris, pH 7.5, 150 mM NaCl) pre-warmed to 70 °C by vortexing and then bath sonicating until the suspension cleared (about 1 h) to produce SUVs. The SUVs were centrifuged for 10 min at 16,000x*g* and 4 °C (DOPC) or 40 °C (DPPC) to remove large particulates. The SUVs were stored under argon at 4 °C and used to prepare bilayers for a maximum of 1 month.

Planar lipid bilayers were prepared by diluting DOPC SUVs to 0.2 mg/ml in PBS, and DPPC SUVs to 0.2 mg/ml in fusion buffer (10 mM Tris, pH 7.5, 300 mM NaCl, 10 mM $MgCl_2$) pre-warmed to 70 °C, and adding 10 µl to a CultureWell gasket attached to a piranha-etched coverslip. SUVs were incubated on the coverslip for 45 min in a sealed humidity chamber at either room temperature (DOPC) or 70 °C (DPPC) to allow vesicles to fuse. The bilayers were washed with PBS (DOPC),

or with fusion buffer followed by PBS (DPPC) to remove unfused vesicles. The bilayers were then incubated sequentially with 100 µg/ml BSA for 1 h, 20 min with 0.5 µg/ml streptavidin, and 10 min with antigen-conjugated DNA sensors, which were titrated to the densities reported in Table S1. Sensor densities were matched to within +/- 30% across all bilayers on the day of each experiment.

To assess bilayer mobility, FRAP measurements were performed on bilayers doped with 0.005 mol % Liss Rhod PE lipids using an A1R+ confocal microscope (Nikon). A circular region of radius ~17 µm was photobleached for 2 s using a 561-nm laser modulated by a galvanometer scanner. Fluorescence recovery was monitored at 30 s intervals using a gallium arsenide phosphide cathode (GaAsP) detector.

Quantification of DNA sensor density on bilayers. The surface density of fluorescent tension sensors was calibrated following the procedure developed by Galush et al. (6). DOPC bilayers were doped with either Liss Rhod PE or Cy5.5 PE lipids at concentrations ranging from 0 to 0.06 mol % or 0 to 0.5 mol %, respectively, and imaged with the same conditions used for cell-based measurements to generate a calibration curve to map fluorescence intensity to number of lipid fluorophores. To use the calibration curve to determine the number of antigen molecules on the bilayer, the sensor fluorescence intensity was compared to the lipid fluorescence intensity to obtain the F factor, defined as: $F = I_{bulk(sensor)}/I_{bulk(lipid)}$, where $I_{bulk(sensor)}$ and $I_{bulk(lipid)}$ are the intensities of DNA tension sensor or lipid in solution at the same concentration. The intensity values were measured in imaging buffer, 2 µm above the glass coverslip.

Fluorescence microscope. TIRF and z-stack images were acquired using a Nikon TiE TIRF microscope equipped with a 100x, 1.49-NA oil-immersion objective (Nikon), a motorized stage with an integrated piezo Z-drive (MS-2000; Applied Scientific Instrumentation), and active Z-drift correction (Perfect Focus System; Nikon). The microscope was controlled by a high-speed TTL, I/O, DAC controller (Triggerscope 4; Cairn Research) integrated into MicroManager software (7). Illumination was supplied by a MultiLine LaserBank (Cairn Research) fitted with 405-, 488-, 561-, and 640-nm diode lasers (Coherent OBIS). The beams were aligned into a single-mode fibre and coupled to an iLas2 Targeted Laser Illuminator (Gataca Systems), which produces a 360° spinning beam with an adjustable illumination angle. Laser beams were passed through a laser quadband (405/488/561/640nm) filter set for TIRF applications (TRF89901-v2-ET; Chroma) before illuminating the sample. Emitted photons were filtered by appropriate single-band emission filters (Chroma) using a filter wheel (OptoSpin; Cairn Research) and then captured onto a back-illuminated sCMOS camera (Prime 95B sCMOS; Teledyne Photometrics). For live-cell imaging, a relative humidity of 95% and a constant temperature of 37 °C was maintained using a cage incubator fitted with an active humidity controller (Okolab).

Single-particle tracking analysis of antigen-DNA diffusion. Bilayers were incubated with a low density of antigen-DNA sensors to enable detection of single particles. The sensors were imaged using 561 nm excitation, with a power density of 18 W/cm^2 at the sample, to visualize the Atto550 fluorophore labelling the DNA. Images were acquired with 10 ms exposure and no delay between frames at 37 °C. Sensors were detected and tracked using the ImageJ plugin TrackMate (8). A Difference of Gaussians filter was used to detect spots of ~0.5 μm diameter using a quality threshold of 2 and sub-pixel localization. Simple LAP tracker was used for tracking, with a 1-μm linking maximum distance, 1.5-μm gap-closing maximum distance, and 2-frame or 20-ms gap-closing maximum gap. Trajectories with 9 or more steps were carried forward for mean-squared displacement (MSD) analysis.

The MSD for each trajectory was calculated using Track Processor MSD in the Track Manager plugin of Icy (9). For a Brownian particle diffusing in two dimensions,

$$\langle r^2 \rangle = 4D\tau^{\alpha}$$

where $\langle r^2 \rangle$ is the mean-squared displacement (μm^2) of the particle during time interval τ (s), D is the diffusion constant (μm^2/s), and α is the anomalous diffusion exponent that defines Brownian ($\alpha = 1$), sub-diffusive ($\alpha < 1$), and super-diffusive ($\alpha > 1$) motion. The diffusion constant was calculated using the first 26 time lags for DOPC and the first 11 time lags for DPPC, where the plots of $\langle r^2 \rangle$ versus τ were linear. Likewise, anomalous diffusion exponents (α) were derived from the initial 5 time lags of the log-log plots of the mean-squared displacement as a function of the time interval.

Fixed-cell imaging. B cells were washed and resuspended in warm Hank's balanced salt solution, calcium, magnesium, no phenol red (HBSS; Gibco) supplemented with 0.1% BSA (HBSS 0.1% BSA). Cells were allowed to recover at 37 °C for 5 min and then added to HBSS 0.1% BSA in pre-warmed imaging chambers with the antigen-coated bilayers and incubated for the indicated timepoints at 37 °C to allow B cells to form immune synapses and extract antigen for internalization. Cells were fixed in 2.6% paraformaldehyde (PFA) for 10 min, blocked with 5% (v/v) normal mouse serum (Jackson ImmunoReserach) for 30 min, and stained with 1 μg/ml anti-mouse/human B220 for 30 min at room temperature or overnight at 4 °C. Stained cells were washed, permeabilized, and then incubated with primary antibodies for intracellular staining (Table S4).

For staining with anti-NF-κB (p65) and anti-NFAT, cells were permeabilized with the FoxP3 fixation/permeabilization kit (BioLegend). For all other intracellular stains, cells were permeabilized with HBSS supplemented with 1% BSA, 5% normal mouse serum, and 0.3% Triton X-100. After washing with permeabilization buffer, cells were incubated with secondary antibodies and phalloidin to stain filamentous actin. Cells were PFA fixed and washed again before imaging either in TIRF or by acquiring z-stacks with a 0.5-μm step size. We note that the fixing process often results in unquenching of the Atto647N signal at the immune synapse, as evident in Figures 7 and

8. This phenomenon does not affect the quantification of internalized clusters, as we only include clusters positioned at least 1.5 μm (3 z-slices) above the synapse plane in our analysis (detailed in "Z-stack image processing"). For unfixed samples, such as those used in live-cell imaging of antigen extraction dynamics (Figure 6 and Movie S3), the IBRQ-mediated quenching of Atto647N exceeds 98% efficiency when the DNA sensor is intact (10).

To measure antigen affinity discrimination, B cells were stimulated on bilayers presenting either NIP_3-DNA or NP_3-DNA complexes. There were four conditions for each experiment: DOPC NIP_3-DNA, DOPC NP_3-DNA, DPPC NIP_3-DNA, and DPPC NP_3-DNA. B cells were stimulated in parallel across all substrates and imaged with the same imaging conditions.

TIRF image processing. Images were cropped to remove poorly illuminated regions, background subtracted, and flatfield-corrected before proceeding to image analysis. Semi-automated ImageJ macros segmented cells based on B220 surface marker staining or, where B220 was not available, the phalloidin staining was used to segment cells. All segmentations were manually assessed, and poorly segmented cells were removed prior to analysis. The resulting 2D masks were used for subsequent analysis. Fluorescence intensities in other channels were calculated from the pixels identified in the cell masks, with quantification of phospho-signaling markers (pSyk, pMLC) and F-actin restricted to the synapse plane. Cell spread area was defined as the area of the cell mask at the synapse plane.

Z-stack image processing. Prior to image processing and analysis, images were cropped, background-subtracted, and flatfield-corrected. Analysis of multi-color z-stack images was performed with a user-guided pipeline in MATLAB (MathWorks) tethered to ImageJ via the MIJ plugin (http://bigwww.epfl.ch/sage/soft/mij/) as previously described (11). Briefly, cells were detected first in 2D and then in 3D using both the B220 and brightfield channels. 3D cell masks were then stored as image stacks for subsequent analysis, excluding any cells touching the edge of the image. The synapse was identified as the sharpest image plane in the Atto550 channel. Antigen extraction was analysed for each cell by bandpass filtering each plane and identifying Atto647N-positive clusters above the synapse using a user-defined global threshold. Antigens were considered internalized if they were at least 1.5 μm above the synapse plane. Local background due to fluorescence scattered from the substrate was subtracted from each identified antigen cluster. Data were inspected and manually gated using a custom-written visualization tool in MATLAB to remove any non-cellular debris, incorrectly segmented cells, and cells poorly stained with anti-B220.

The mean and total Atto550 fluorescence intensity of the pixels in the synapse plane were used as a measure of synaptic antigen binding. The total extracted antigen was calculated as the sum of Atto647N pixel intensities in all extracted antigen clusters. The efficiency of antigen

extraction was calculated as the total extracted antigen intensity (Atto647N) divided by the total synaptic antigen intensity (Atto550). The mean Atto647N intensity of extracted clusters was used as a measure of antigen per cluster.

Masks of the extracted antigen clusters, synapse, and total cell were used to quantify fluorescence in other channels. To identify antigens within MHCII+ compartments, we defined a threshold based on manual assessment of a representative subset of cells. The analysis revealed that antigen clusters within MHCII+ compartments exhibited MHCII intensity at least 25% higher than the mean endosome MHCII intensity across all cells. This threshold was applied to calculate the percentage of internalized clusters located within MHCII+ compartments.

Cell spread area was calculated from the area of the cell mask at the synapse plane. Cells were considered to have failed to extract antigen if no antigen clusters were detected in the cell.

Transcription factor translocation. B cells were stimulated with activating bilayers for 1 h at 37 °C, fixed and stained with anti-B220 to label the cell surface, permeabilized and stained with either anti-NF-kB (p65) or anti-NFAT and DAPI, and imaged at a single plane approximately 2 µm above the bilayer surface where the DAPI staining was sharpest. Images were analysed in CellProfiler (12). For each cell, the nucleus and cytosol were identified from the DAPI and B220 channels and masked as different regions, and the transcription factor mean intensity quantified in each region. The nuclear translocation for each cell was calculated as the ratio of nuclear-to-cytosolic mean intensity.

Live-cell calcium imaging and analysis. B cells were loaded with 5 µM Cal-520 AM (AAT Bioquest) at 37 °C in full RPMI for 1.5-2 h at a density of 5 x 10^6 cells/ml. After 1.5-2 h, cells were washed into fresh RPMI and stained at the surface with Alexa Fluor 405 anti-IgM Fab (Jackson ImmunoResearch). For Fab staining, cells were stained for 30 min on ice at a density of 6 x 10^6 cells/ml, with mixing by pipetting every 10 min (13). Cells were pelleted and resuspended in warm HBSS 0.1% BSA and allowed to recover for 5 min at 37 °C immediately before imaging. Cells were imaged on sensor-coated bilayers for at least 10 min by time-lapse TIRF microscopy, with images acquired every 8 s.

For analysis, cells were segmented using the anti-IgM Fab stain using K-means thresholding in a custom Icy pipeline to obtain the cell area and background-subtracted intracellular calcium intensity over time. Calcium traces were normalized to the first frame that the cell was in contact with the bilayer and were categorized based on the following rules. If the calcium intensity did not reach at least double the intensity value in frame 1, it was considered “non-responsive”. Otherwise, a line was drawn at the full width at half maximum (FWHM) for the first Ca^{2+} peak. If the calcium trace crossed the line twice, resulting in a single peak, it was categorized as “unique”; if the trace crossed the line four or more times, resulting in at least two peaks, it was categorized as

"oscillatory"; if the trace crossed the line once and remained above it for the duration of the measurement, it was categorized as "sustained".

Live-cell imaging and analysis of antigen extraction. B cells were seeded onto sensor-coated bilayers and imaged sequentially in the 561-nm and 640-nm channels to visualize synaptic and extracted antigen, respectively. Cells were imaged for at least 10 minutes with images acquired every 8 s. The Atto647N fluorophore remains quenched in the synapse by Iowa Black RQ, and is unquenched only upon DNA tether rupture, indicating antigen extraction. Extracted antigen clusters were identified in widefield as spots having an intensity at least 1.5x the background Atto647N intensity within the cell.

For each experiment, 6 representative cells that internalized antigen from DOPC and DPPC substrates and had sharp antigen spots were selected for detailed analysis. A maximum projection of the anti-IgM Fab signal was used to segment the cell using default thresholding in ImageJ. The cell center of mass was then obtained using the Analyze Particles function. TrackMate (8) was used to detect and track the position and intensity extracted antigen clusters in the Atto647N channel. The xy coordinates of the first point in each trajectory were taken to be the position of antigen extraction and were used to calculate the distance from the cell center of mass. The time point of antigen extraction was taken to be the time between when the cell first contacted the bilayer and the time when the extracted antigen cluster appeared.

To bin extracted antigen clusters into the central, peripheral, or distal regions, at each time point in a live-cell video each cell was divided into three concentric circles of equal width centered around the cell center of mass, which was identified based upon the anti-IgM surface stain. Extracted clusters were assigned to a bin based upon the xy coordinates of the frame in which they first appear.

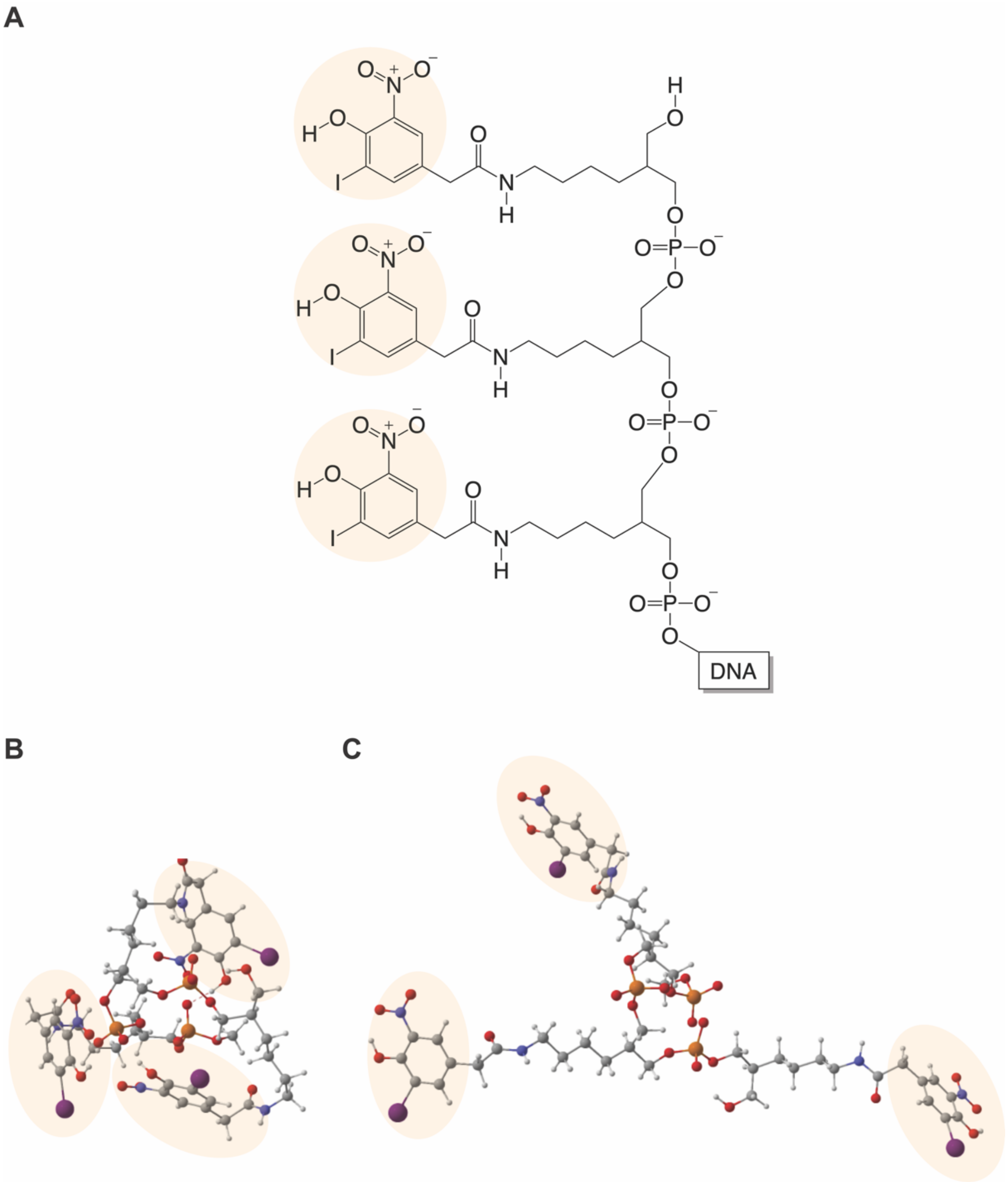

Fig. S1. (A) Molecular structure of NIP_3 antigen. (B, C) To compute the structures, the DNA molecule indicated in (A) was replaced with a methyl group. (B) The CREST-optimized structure used to estimate the equilibrium (minimum) inter-hapten distance. (C) The fully extended structure used to estimate the maximum inter-hapten distance. Atom color-coding: gray-carbon; white-hydrogen; blue-nitrogen; red-oxygen; orange-phosphorus; purple-iodine. Hapten moieties are highlighted with yellow circles.

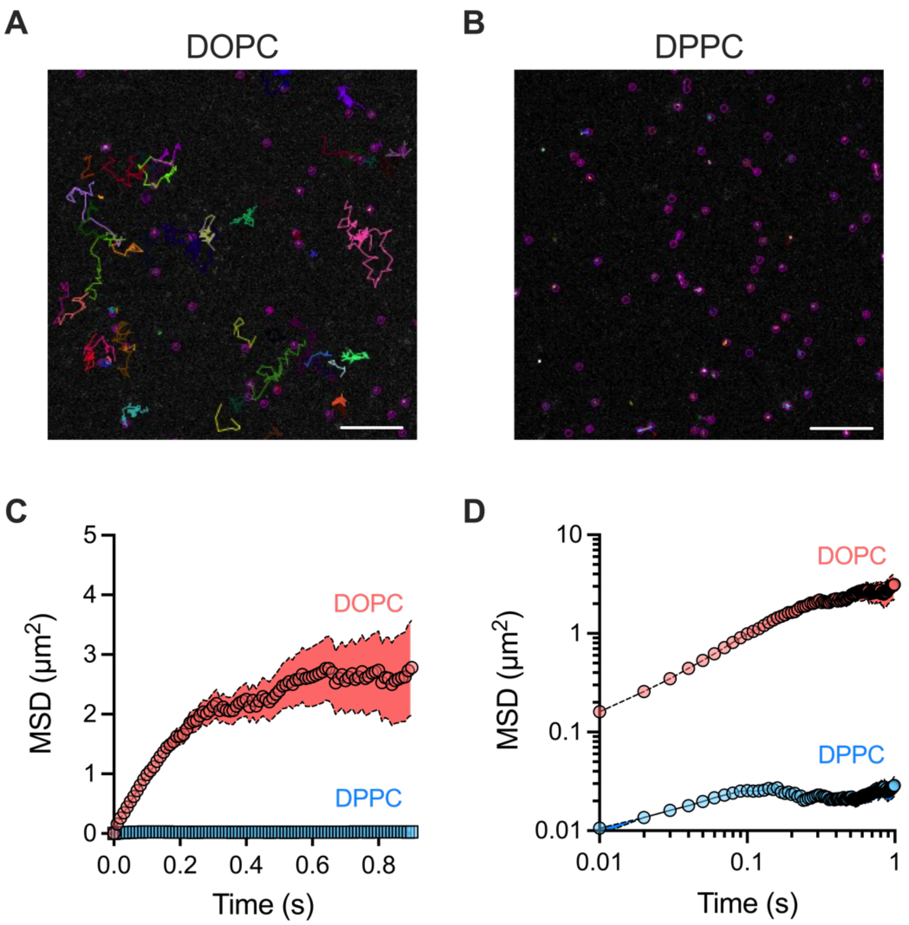

Fig. S2: Diffusion dynamics of DNA sensors on DOPC and DPPC planar lipid bilayers. (A, B) Single-particle tracking of fluorescent DNA sensors on bilayers. Pink circles indicate detected particles, and colored lines show their trajectories overlaid on raw fluorescence images of DOPC (A) and DPPC (B) bilayers. Scale bars: 5 μm. (C) Time evolution of the average mean square displacement (MSD). Diffusion coefficients were calculated from the linear regions of MSD versus time curves, spanning the first 26 and 11 time lags for DOPC and DPPC, respectively. (D) Mean MSD plotted against time on logarithmic scales for both bilayers. Anomalous diffusion exponents (α) were derived from the initial 5 time lags of the log-log MSD plots. Analysis encompasses 3 independent experiments, comprising 673 tracks on DOPC and 1,857 tracks on DPPC bilayers.

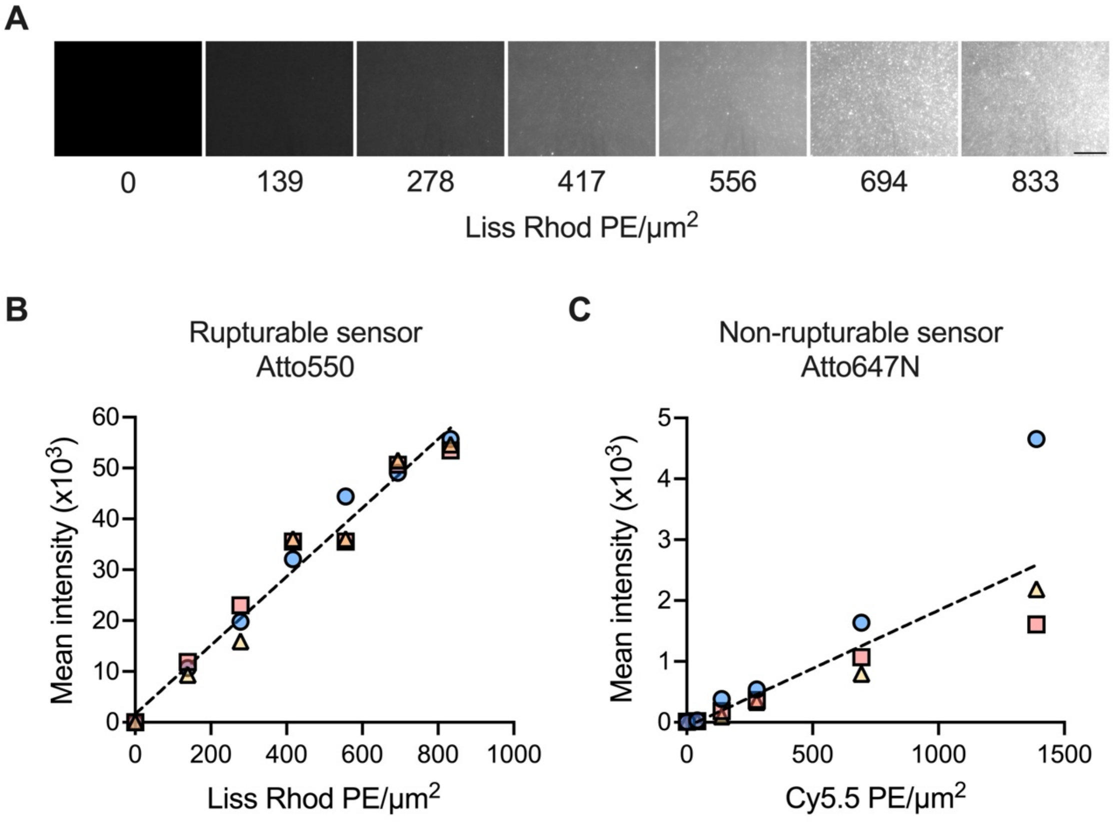

Antigen	F factor (mean ± SD)
NIP_3	0.057 ± 0.008
NP_3	0.069 ± 0.005

Antigen	F factor (mean ± SD)
NIP_3	0.261 ± 0.014
NP_3	0.243 ± 0.113

Fig. S3: Calibration of fluorescence intensity for molecular density measurements. (A) Representative fluorescence microscopy images of DOPC lipid bilayers doped with increasing amounts of Liss Rhodamine PE lipids. Surface densities of fluorescent lipids are indicated for each bilayer. Scale bar: 10 μm. Image credit: reprinted with permission from ref. (1). (B, C) Top panels show calibration curves derived from 3 independent bilayer measurements using (B) Liss Rhodamine PE lipids and (C) Cy5.5 PE lipids. Bottom panels show the F factor values comparing fluorescence intensities of (B) the Atto550 fluorophore on the rupturable tension sensor to Liss Rhodamine PE, and (C) Atto647N fluorophore on the non-rupturable DNA sensor to Cy5.5 PE. F factors were determined separately for sensors conjugated to NIP_3 and NP_3 antigens. Image credit: plot in (B) reprinted with permission from ref. (1).

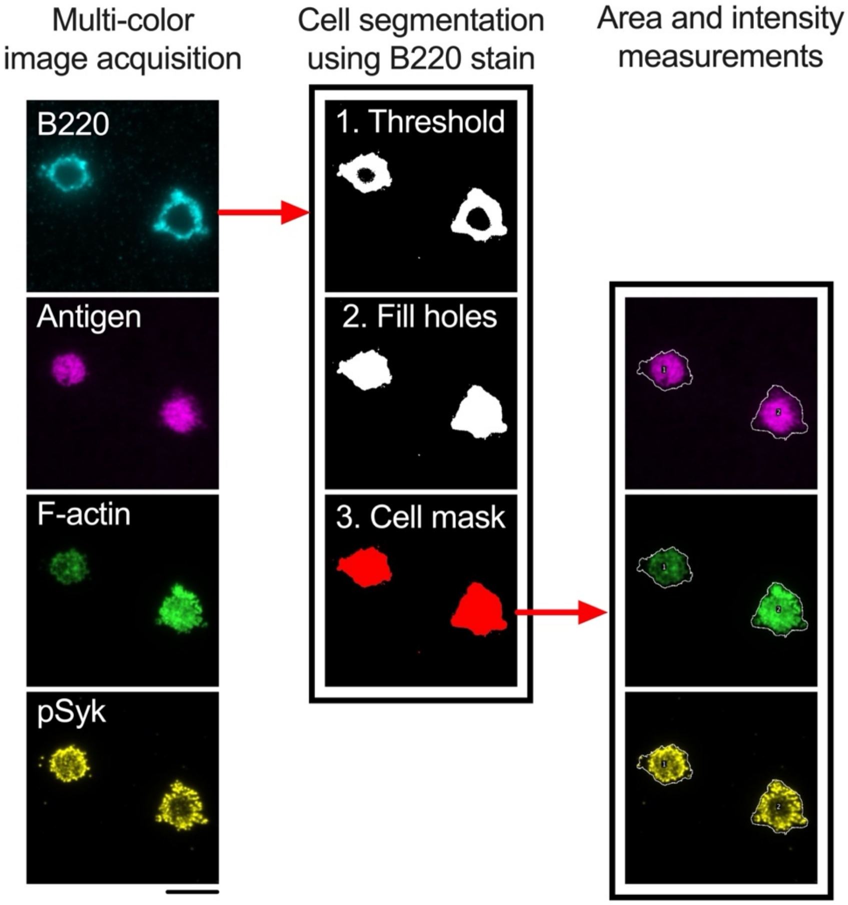

Fig. S4: Cell segmentation workflow for TIRF microscopy image analysis. Multi-color TIRF images are processed in Fiji by segmenting cells in the B220 channel. Segmentation involves applying a default threshold, filling holes, and generating cell masks using the Analyze Particles function (parameters: size 10-10,000 μm^2, circularity 0.20-1.00). The resulting mask defines cell boundaries for area measurements and quantification of fluorescence intensities across all channels. Scale bar: 10 μm.

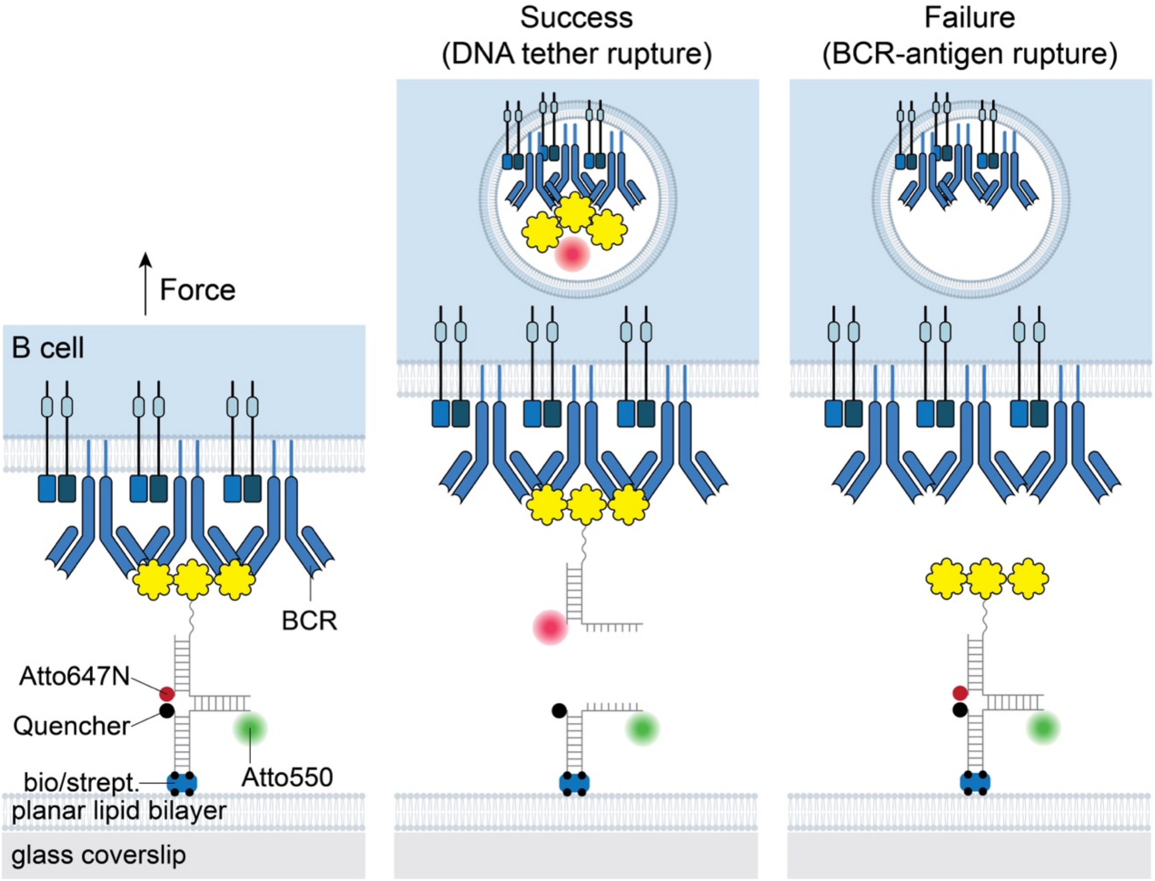

Fig. S5. Schematic of the DNA tension sensor used to quantify force-mediated antigen extraction. The DNA sensor releases the antigen if the B cell applies mechanical forces higher than ~10 pN to the BCR-antigen bond (10).

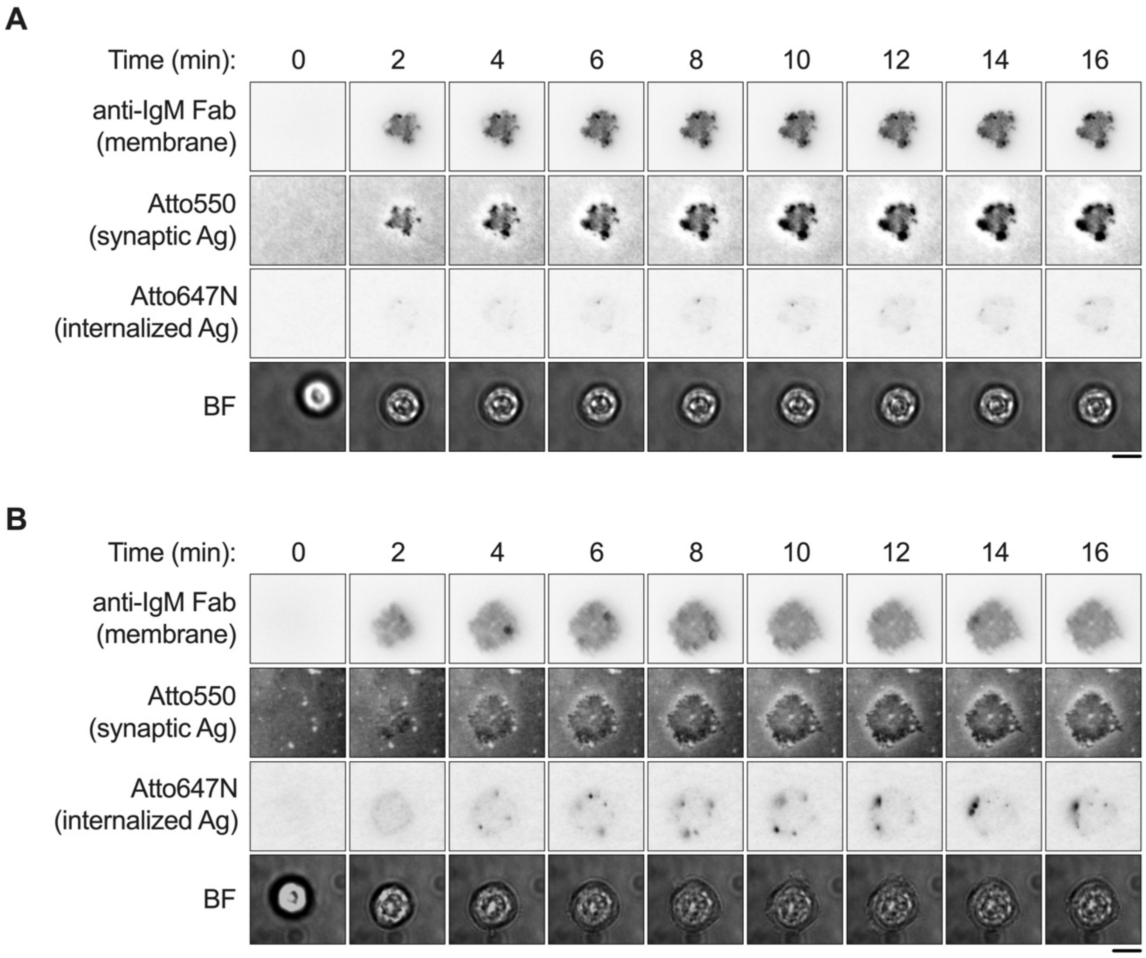

Fig. S6. Still images of B cell antigen extraction from DOPC (A) and DPPC (B). Extracted clusters appear as dark spots in the Atto647N channel. Scale bars: 5 μm.

Table S1. Antigen densities and sensors used for each experiment. The average surface density of antigen-DNA complexes across all experiments was 2870 ± 1506 (mean ± SD).

**Measurement**	**Figure**	**Antigen-DNA complexes/μm^2 (mean ± SD)**	**Sensor**
Cell spreading, antigen mean and total intensity, and pMLC intensity	2	6287 ± 3241	Non-rupturable
pSyk and F-actin intensity	2	8043 ± 1340	Non-rupturable
Calcium flux	3	999 ± 184	Rupturable
Transcription factor activation	4	3217 ± 2287	Rupturable
Fixed-cell antigen extraction	5	1773 ± 511	Rupturable
Live-cell antigen extraction	6	989 ± 184	Rupturable
Antigen-MHCII colocalization	7	2401 ± 579	Rupturable
Antigen affinity discrimination	8	1120 ± 1111 (NIP_3-DNA)<br>1003 ± 998 (NP_3-DNA)	Rupturable

Table S2. Oligonucleotide sequences used to make the non-rupturable DNA sensor.

Strand name	Sequence (5'-3')
49 bp	Uni-Link Amino modifier (UniAmM)-UniAmM-UniAmM/TCA CGA CAG GTT CCT TCG CAT CGA TAC ATG TGT GAC GAA CAC TCA TTT A/Biotin
49 bpC	Biotin/TAA ATG AGT GTT CGT CAC ACA TGT ATC GAT GCG AAG GAA CCT GTC GTG A/Atto647N

Table S3. Oligonucleotide sequences used to make the rupturable DNA sensor. Color coding: red—24-bp upper handle; purple: 24-bp lower handle; blue: 20-bp tether.

**Strand name**	**Sequence (5’-3’)**
UH24 20R20	UniAmM-UniAmM-UniAmM/ TCA CGA CAG GTT CCT TCG CAT CGA TAT TCT ATG TTA CTA TGT AA
20R20C LH24	Atto550/ TTA CAT AGT AAC ATA GAA TAA CAT GTG TGA CGA ACA CTC ATT TA/Biotin
UH24C	Atto647N/ TCG ATG CGA AGG AAC CTG TCG TGA
LH24C	Biotin/ TAA ATG AGT GTT CGT CAC ACA TGT/Iowa Black RQ

Table S4. Antibodies and probes used for fluorescence staining.

Antibody	Clone	Dilution or final conc.	Incubation time (min)	Supplier	Cat. No.
IgM Fab AF405		10 µg/ml	30 min on ice	Jackson (labeled in-house)	115-007-020
CD45R/B220 BV421	RA3-6B2	1 µg/ml	30 min at RT or o/n at 4 °C	BD	562922
CD45R/B220 PerCP/Cy5.5	RA3-6B2	1 µg/ml	30 min at RT or o/n at 4 °C	BioLegend	103236
MHC II (I-A/I-E) AF488	M5/114.15.2	1 µg/ml	30	BD	562352
NFAT1		1:50	60	Cell Signaling	4389S
NF-κB (p65)	D14E12	1:400	60	Cell Signaling	8242S
p-BLNK (pY84) AF488	J117-1278	1:20	60	BD	558444
p-Myosin Light Chain 2	T18/S19	1:200	60	Cell Signaling	3674S
p-Syk (Y525/526)	C87C1	1:50	30	Cell Signaling	2710S
Anti-mouse IgG (H+L) F(ab')$_2$ AF488		1:2000	60	Cell Signaling	4408S
Anti-rabbit IgG (H+L) F(ab')$_2$ AF488		1:2000	60	Cell Signaling	4412S
Anti-rabbit IgG (H+L) F(ab')$_2$ AF555		1:2000	60	Cell Signaling	4413S
Anti-rabbit IgG (H+L) F(ab')$_2$ AF647		1:2000	60	Cell Signaling	4414S
Phalloidin AF488		1:200	60	Invitrogen	A12379
DAPI ready-made solution		1:1000	60	Sigma-Aldrich	MBD0015

Movie legends

Movie S1. Single-particle imaging of antigen-DNA complexes on DOPC. Images were acquired with 10 ms exposure time and 0 s between frames.

Movie S2. Single-particle imaging of antigen-DNA complexes on DPPC. Images were acquired with 10 ms exposure time and 0 s between frames.

Movie S3. Live-cell imaging of antigen extraction from bilayer substrates. Dark spots appear when B cells rupture antigens from the DNA tether, separating an Atto647N fluorophore from a dark quencher. Extraction from DOPC is shown on the left, and from DPPC on the right. Images were acquired every 8 s. Scale bar: 5 µm.

References

1. H. C. W. McArthur, A. T. Bajur, K. M. Spillane, Quantifying force-mediated antigen extraction in the B cell immune synapse using DNA-based tension sensors. *Methods Cell Biol.* **193**, 99-126 (2025).
2. M. D. Hanwell *et al.*, Avogadro: an advanced semantic chemical editor, visualization, and analysis platform. *J. Cheminform.* **4**, 17 (2012).
3. C. Bannwarth, S. Ehlert, S. Grimme, GFN2-xTB-An Accurate and Broadly Parametrized Self-Consistent Tight-Binding Quantum Chemical Method with Multipole Electrostatics and Density-Dependent Dispersion Contributions. *J. Chem. Theory Comput.* **15**, 1652-1671 (2019).
4. P. Pracht, F. Bohle, S. Grimme, Automated exploration of the low-energy chemical space with fast quantum chemical methods. *Phys. Chem. Chem. Phys.* **22**, 7169-7192 (2020).
5. P. M. Nair, K. Salaita, R. S. Petit, J. T. Groves, Using patterned supported lipid membranes to investigate the role of receptor organization in intercellular signaling. *Nat. Protoc.* **6**, 523-539 (2011).
6. W. J. Galush, J. A. Nye, J. T. Groves, Quantitative fluorescence microscopy using supported lipid bilayer standards. *Biophys. J.* **95**, 2512-2519 (2008).
7. A. Edelstein, N. Amodaj, K. Hoover, R. Vale, N. Stuurman, Computer control of microscopes using microManager. *Curr. Protoc. Mol. Biol.* **Chapter 14**, Unit14 20 (2010).
8. J. Y. Tinevez *et al.*, TrackMate: An open and extensible platform for single-particle tracking. *Methods* **115**, 80-90 (2017).
9. F. de Chaumont *et al.*, Icy: an open bioimage informatics platform for extended reproducible research. *Nat. Methods* **9**, 690-696 (2012).
10. K. M. Spillane, P. Tolar, B cell antigen extraction is regulated by physical properties of antigen-presenting cells. *J. Cell Biol.* **216**, 217-230 (2017).
11. C. R. Nowosad, K. M. Spillane, P. Tolar, Germinal center B cells recognize antigen through a specialized immune synapse architecture. *Nat. Immunol.* **17**, 870-877 (2016).
12. D. R. Stirling *et al.*, CellProfiler 4: improvements in speed, utility and usability. *BMC Bioinformatics* **22**, 433 (2021).
13. A. Droubi *et al.*, The inositol 5-phosphatase INPP5B regulates B cell receptor clustering and signaling. *J. Cell Biol.* **221**, e202112018 (2022).
